# How our longitudinal employment patterns might shape our health as we approach middle adulthood—US NLSY79 cohort

**DOI:** 10.1371/journal.pone.0300245

**Published:** 2024-04-03

**Authors:** Wen-Jui Han

**Affiliations:** Silver School of Social Work, New York University, New York, NY, United States of America; Hitosubashi University, JAPAN

## Abstract

Recent labor market transformations brought on by digital and technological advances, together with the rise of the service economy since the 1980s, have subjected more workers to precarious conditions, such as irregular work hours and low or unpredictable wages, threatening their economic well-being and health. This study advances our understanding of the critical role employment plays in our health by examining how employment patterns throughout our working lives, based on work schedules, may shape our health at age 50, paying particular attention to the moderating role of social position. The National Longitudinal Survey of Youth-1979 (NLSY79), which has collected 30+ years of longitudinal information, was used to examine how employment patterns starting at ages 22 (n ≈ 7,336) might be associated with sleep hours and quality, physical and mental functions, and the likelihood of reporting poor health and depressive symptoms at age 50. Sequence analysis found five dominant employment patterns between ages 22 and 49: “mostly not working” (10%), “early standard hours before transitioning into mostly variable hours” (12%), “early standard hours before transitioning into volatile schedules” (early ST-volatile, 17%), “mostly standard hours with some variable hours” (35%), and “stable standard hours” (26%). The multiple regression analyses indicate that having the “early ST-volatile” schedule pattern between ages 22 and 49 was consistently, significantly associated with the poorest health, including the fewest hours of sleep per day, the lowest sleep quality, the lowest physical and mental functions, and the highest likelihood of reporting poor health and depressive symptoms at age 50. In addition, social position plays a significant role in these adverse health consequences. For example, whereas non-Hispanic White women reported the most hours of sleep and non-Hispanic Black men reported the fewest, the opposite was true for sleep quality. In addition, non-Hispanic Black men with less than a high school education had the highest likelihood of reporting poor health at age 50 if they engaged in an employment pattern of “early ST-volatile” between ages 22 and 49. In comparison, non-Hispanic White men with a college degree or above education had the lowest likelihood of reporting poor health if they engaged in an employment pattern of stable standard hours. This analysis underscores the critical role of employment patterns in shaping our daily routines, which matter to sleep and physical and mental health as we approach middle adulthood. Notably, the groups with relatively disadvantaged social positions are also likely to be subject to nonstandard work schedules, including non-Hispanic Blacks and people with low education; hence, they were more likely than others to shoulder the harmful links between nonstandard work schedules and sleep and health, worsening their probability of maintaining and nurturing their health as they approach middle adulthood.

## Introduction

Since the 1980s, the rise of the technological and digital age has transformed how people around the globe live and work, carrying significant consequences for our overall well-being [1]. For instance, innovations in medicine and public health have increased life expectancy in the United States from 48 years in 1900 to 76 years in 2000 [2]. However, since the 1990s, health improvements might have been blunted by the increased prevalence of precarious employment [3]. Precarious jobs are defined as those with poor working conditions and weak power relations, including low wages, unpredictable or unstable hours, few or no benefits, and weak or no bargaining power. One of the essential indicators of a precarious job is working outside of traditional 9:00 to 5:00 hours, such as during early mornings, evenings, or nights, or having irregular hours (e.g., rotating, split, or unpredictable hours). Such work patterns are sometimes called nonstandard work schedules [4] or shiftwork [5]. Prior research has found that these jobs have physically exhausted and emotionally drained U.S. workers [3]. Recently, the COVID-19 crisis has heightened existing inequalities, as people engaging in shiftwork (ironically labeled “essential” work) experience greater exposure to infection and higher death tolls [6].

Approximately one-third of the workforce globally has a work schedule considered nonstandard or shiftwork [7]. Dr. Harriet Presser [8] was among the first to extensively document this labor force transformation. Her seminal works provide insights into not only the prevalence of such work schedules but also their potential implications for the well-being of individuals and their families [4, 8]. Subsequent studies have demonstrated that nonstandard work schedules dictate when we can sleep, with implications for our sleep quality and overall physical and mental health [5]. Studies using samples from different occupations (e.g., nurses, truck drivers) [9, 10] and countries (e.g., Canada, European countries, Singapore, South Korea, and the United States) [1, 5, 11–13] have consistently documented significantly worse health outcomes, including shorter sleep and a lower quality of sleep, among those with nonstandard work schedules compared to their counterparts. A growing line of scholarship has also documented well-established adverse associations between nonstandard work schedules, particularly night shifts, and a higher likelihood of poor physical health (e.g., cardiovascular disease) and mental health (e.g., anxiety, depression) [1, 5, 8].

Missing from the extant scholarship are longer term longitudinal studies using a life-course perspective with sequence analysis to examine how work schedule patterns might be associated with our sleep and health as we approach middle adulthood [14]. This study extends our knowledge by using the National Longitudinal Study of Youth-1979, a nationally representative sample of about 7,000 people in the U.S. over 30 years, from ages 22 to 50. I focus on work schedule patterns in the United States throughout individuals’ working lives to underscore the critical role of employment in our daily experience and thus our health. This study also fills a literature gap by paying attention to how such a link might differ by social position, as reflected by race-ethnicity, gender, and education. This study, therefore, provides new insights into factors shaping our well-being on a global scale given that nonstandard work schedules are increasingly becoming a global phenomenon [15].

### Life-course approach using a cumulative advantage and disadvantage (CAD) lens

This study builds on the life-course perspective [16, 17] to conceptualize the association between employment throughout adulthood and sleep and health at age 50. Specifically, firstly, drawing on a fundamental principle of the life-course perspective—that human development and aging are lifelong processes, with the appreciation that the past shapes the future—this study uses longitudinal data to conceptualize and empirically examine how work patterns during one’s working life between ages 22 and 49 may shape sleep and physical and mental health at age 50. Importantly, our health is shaped by daily events occurring to and around us but may not manifest their effects until years later. Hence, studying working lives over substantial periods allows one to identify and investigate long-term associations between changes in our employment concerning work schedules and our health. For example, we can never be certain that no association between employment patterns and our sleep and health exists merely based on short-term null effects. Building upon this principle, this study answers the following research question: How might lifetime work trajectories shape future health outcomes? Secondly, drawing on the principle of timing—that the health consequences of event transitions and patterns vary according to their timing in a person’s life—this study uses longitudinal data spanning more than 30 years of an individual’s life to understand how transitions between work schedules over time may shape sleep and health at age 50. By examining work trajectories, this study, thus, pays attention to the paths of changes in individuals’ employment patterns and transitions that might shape their health, taking a long view of the life course. Building upon this principle, this study also answers the following research question: How might transitions between schedules (for example, daytime hours to evening or night hours) be associated with our sleep (hours and quality) and our future physical and mental health? Overall, examining the constraints imposed by employment patterns, particularly work schedules, with a life-course lens allows us to understand how favorable work conditions from early adulthood to old age contribute to better health in an individual’s lifetime, with significant implications for the well-being of future generations.

Furthermore, I pay special attention to how the links between employment patterns throughout one’s working life and sleep health at age 50 might vary by social position, identified in this study through race-ethnicity, gender, and education. I adopt the cumulative advantage and disadvantage (CAD) framework [18, 19], which assumes our social positions (e.g., race, ethnicity, gender) interact with macro systems and institutions (e.g., employment) to shape our opportunities and constraints throughout our lifetime, influencing our health by generating increasing disparities in resources between those who have and those who have not [16, 18, 19]. Importantly, some work schedules (e.g., daytime hours) are more likely than others (e.g., irregular hours) to benefit our sleep and health [1, 5]. By considering social position in this investigation, I shed light on the prevalent health disparities among different social position groups that might partly result from work schedule patterns over time.

### Health consequences of work schedules

Since the late 1990s, studies using both US and non-US samples have examined the links between work schedules and social, psychological, and physical well-being of individuals [1, 8, 20] and family members, including children [4, 21, 22]. These studies have largely found weak to moderate adverse associations between working nonstandard hours and the well-being of workers and their families, particularly when such a schedule was chosen involuntarily (e.g., a job requirement). One of the immediate adverse health consequences is a decline in the amount and quality of sleep for workers with nonstandard hours because these schedules (e.g., night shifts) counter our circadian rhythm, which is critical for maintaining and sustaining good health [5]. Health issues stemming from severe sleep deprivation and low sleep quality due to nonstandard work schedules have been labeled Shift Work Sleep Disorder (SWSD) by academics and experts in the medical field [5]. People with SWSD tend to report the following symptoms: trouble sleeping, excessive sleepiness, and tiredness. These symptoms compromise one’s overall physical and mental functions, leading to poor general health [5]. Regarding other health consequences, one study showed that 38% of people working an 8-hour night shift had a BMI ≥30 versus 26% of people working an 8-hour day shift (*p* < .05) [23]. Another study found that people with nonstandard work schedules are also 42% more likely to suffer from depressive symptoms than those with standard schedules [24].

Our understanding of the links between work schedules and sleep and health has also been refined through increasingly sophisticated data, including from small cross-sectional samples [12, 25], nationally representative samples [1, 26], and panel data [27, 28]. For example, using two-year longitudinal data on approximately 1,500 Norweigan nurses, Waage and colleagues found that nurses working night hours the prior year were more likely to report acute sleepiness or insomnia related to shiftwork in the current year [27]. Importantly, those who stopped working night shifts were more likely to report a reduction in excessive sleepiness and less insomnia. A recent study using panel data from 2002 to 2018 in Germany found that individuals who perceived their work as involving nonstandard work schedules, high job insecurity, and low social rights were more likely to have poorer physical and mental health than their counterparts, and chronic exposure to or transitioning into such work might predict poorer health than otherwise [28]. This study builds on this emerging literature to advance our knowledge by using sequence analysis to document the changes in work trajectories and then examining how those changes/trajectories might be associated with sleep and health.

#### The importance of social position

Another line of studies has shown that social position shapes our likelihood of having jobs requiring nonstandard work schedules [3, 8]. For example, Presser extensively documented that in the United States, young workers, Blacks, and people with a high school or lower education are particularly subject to working nonstandard schedules [8]. In addition, whereas men are more likely than women to have nonstandard schedules, the distribution can vary greatly by occupation. Nurses are a prime example of a female-dominated occupation requiring nonstandard work schedules, particularly night shifts. A substantial line of scholarship has documented that, compared to their counterparts, female workers with nonstandard work schedules, particularly night shifts, have substantially higher odds of experiencing sleep disturbance and fatigue [26], stroke [29], and breast cancer [30]. In addition, studies have shown that both shift work and being an African American independently increase the odds of having high blood pressure [31]. A growing body of evidence has also found that people of racial-ethnic minority groups are more likely to get insufficient or low-quality sleep. Adverse sleep issues, such as insomnia, may also help account for increasing health disparities, such as higher rates of cardiovascular disease among racial-ethnic minority groups [32]. The higher share of African Americans with jobs requiring nonstandard schedules than their counterparts does not help and may indeed further intensify the high prevalence of sleep issues among people of color.

Hence, employment carries long-lasting implications for the social, psychological, physical, and economic well-being of workers and their families, with significant implications for inequality across generations, a central CAD tenet. The rise in precarious jobs, particularly among those in relatively disadvantaged social positions, motivates the need to investigate whether engaging in nonstandard work schedules over time may translate into long-term health consequences. A previous study [33] using the same data as the current analysis found that individuals in various social positions such as men, Blacks, and people with low educational attainment (e.g., high school or less) were more likely to have ever worked nonstandard hours between ages 18 and 39 than were their corresponding counterparts. Importantly, compared to men, women were more likely to have either never or always had nonstandard work schedules by age 39. This finding reflects the reality that women-dominated occupations often require nonstandard hours (e.g., nurses, home health aides).

### The present study

The field has established a decent set of scholarship on the associations between work schedules and sleep and health, including the consequences for family and child well-being. The implication is thus clear. People in the U.S. and around the world are increasingly subject to nonstandard work schedules, creating work-induced health disparities [1, 3, 4, 12]. However, we have yet to understand how employment patterns over the life-course may shape our health as we approach middle adulthood. Furthermore, a long line of extant research has shown how some social positions may act as vulnerabilities, putting people on a disadvantaged trajectory throughout their lifetime [3, 17, 20]. Hence, drawing upon the CAD framework, this study pays attention to three markers representing social position—race-ethnicity, gender, and education—to highlight how the intersectionality between employment and social position may accumulate advantages and disadvantages throughout a lifetime, manifested in our sleep behaviors and general health. By using a nationally representative sample of youths aged 14–22 in 1979 in the United States, this analysis addresses this evidence gap by building upon the life-course and CAD lens to answer the following research questions: how might lifetime work trajectories (between ages 22 and 49) be associated with health outcomes as we approach middle adulthood (at age 50), and how might such an association differ due to the intersectionality between work and social position? Notably, the longitudinal data used in this analysis allow researchers to track employment patterns during a period when working nonstandard hours was on the rise [7, 12].

## Materials and methods

### Data

This study used the National Longitudinal Survey of Youth-1979 (NLSY79), which comprises a nationally representative sample of Americans between the ages of 14 and 22 in 1979 (N = 12,686). NLSY79 interviewed respondents every year until 1994 and biennially thereafter. The current analysis excludes two discontinued oversamples: non-Black non-Hispanic disadvantaged youths, discontinued in 1990 (n = 1,643), and military youth, discontinued in 1984 (n = 1,280). A total of 9,763 respondents served as the starting point after excluding these two discontinued oversamples. The response rates of NLSY79 have been remarkably high, ranging from 96% in the early survey years to about 77% in recent years [34, 35]. The NYLS79 is well suited to this study due to its rich data on longitudinal sociodemographic characteristics (e.g., education, marriage, number of children) and work schedules.

I use outcome measures at age 50 and begin the sample at age 22. I chose age 50 for two primary reasons. First, as this study was built upon a life-course lens, focusing on age 50 allowed me to examine employment patterns over an extended period of time, from ages 22 to 49. Second, NLSY79 collects health outcomes in the health modules at ages 40, 50, and 60. By 2018 (the most updated data as of this analysis), most participants had reached age 50 but only a small proportion (10%) had reached age 60. Therefore, using age 50 meant the sample comprised the majority of the participants. I also had two primary reasons for selecting age 22 as the starting point for the employment patterns. First, NLSY79 did not collect information on ages 14–18 for those who were 19–22 in 1979, the first interview year. Second, more than 30% of the NLSY79 participants were in college between ages 18 and 22. During this period, their jobs, if they had one, were more likely to be temporary or part time [33]. Therefore, age 22 is a plausible beginning point for establishing a career for many participants, particularly the college graduates in the sample.

### Participants

The final analysis excluded participants who were missing information on the sleep outcome at age 50 (n = 2,052) or on employment between ages 22 and 49 (n = 6). Furthermore, approximately 5% of cases (n = 369) were missing information on sociodemographic characteristics (e.g., from < 0.01% on education to about 4% on parental education). The final analyzed samples after these exclusions were 7,336 for the dependent variable of sleep quality, 7,324 for average sleep hours per day over a week, 7,334 for general health status, 7,262 for physical and mental functions, and 7,271 for depression symptoms. Following previous research, missing values for the dependent variables were not imputed to avoid measurement noise [36]. The pattern of missing values on these dependent variables suggests that the older participants (e.g., 19 or older in 1979) were more likely than the younger participants to have missing values on the dependent variables. This missing pattern suggests a positive selection bias; younger or healthier participants more likely to remain in the longitudinal study. No other significant differences in sociodemographic variables were found between those with and without missing outcome measures.

### Measures

#### Sleep

*Hours*. As part of the age 50+ health modules, the NLSY79 asked how many hours of sleep the participant typically gets at night on a weekday, and a separate question asked about the weekend. Using both questions, I created a new variable to represent the average number of hours a participant gets per day across a 7-day week. As a robustness check, the analysis was also run using three individual variables as the outcomes: average number of hours of sleep on a weekday, average number of hours of sleep on the weekend, and average number of hours of sleep per day across a 7-day week. The results were similar to those presented here. Note that information about sleep was not collected before the participants turned 50.

*Quality*. As part of age 50+ health modules, participants were asked how frequently they had experienced the following four issues over the last month: “have trouble falling asleep,” “wake up and have trouble falling back asleep,” “wake up too early and have trouble falling back asleep,” and “feel unrested during the day despite the amount of sleep.” Respondents answered using a 4-point Likert scale ranging from “almost always (4+ times per week)” to “rarely or never (once a month or less).” These four questions are commonly used in studies examining sleep quality or disturbance [37]. A standardized score with a mean of 0 and a standard deviation of 1 was created from these four questions with excellent reliability (α = 0.84). The higher the score, the better the sleep quality was.

#### Poor health

The NLSY79 collects information on general health status by asking participants to assess their general health, ranging from excellent (1) to poor (5). I created a dichotomous variable that received a value of 1 if the participant reported having either “poor” or “fair” health, and 0 otherwise.

#### SF12 physical and mental health

The NLSY79 adopted the 12-Item Short-Form Health Survey (SF-12 v1) to rate self-reported mental and physical health. The NLSY79 administered this scale as part of the 50+ health modules to those who had turned 50 since their last interview. These data were collected between the interview years of 2008 and 2016. Specifically, the respondents were asked 12 questions about the past 4 weeks, including whether pain had interfered with normal work, whether their health had limited their moderate activities, and their frequency of feeling downhearted or blue. The possible responses, given the nature of the question, include a 3-point Likert scale (not limited at all, limited a little, limited a lot) and a 5-point Likert scale (ranging from “all the time” to “none of the time”). This study used the global scores representing physical and mental functions created by the NLSY79, following the scoring established by Ware, Kosinski, and Keller [38]. The SF-12 has been shown to have good reliability (e.g., 0.89) and validity [38] and can detect active and recent depressive disorders [39]. NLSY79 standardized the scores to have a mean of 50 and a standard deviation of 10; a score of 50 corresponds to the U.S. average, and a one-point difference is one-tenth of a standard deviation [40]. Previous research has shown that the NLSY79 sample tends to have a higher-than-average score on SF-12 mental function and just about the average score on SF-12 physical function [40]. The higher the score, the better the function is.

#### Depressive symptoms

As part of the age 50+ health modules, NLSY79 used seven items from the Center for Epidemiologic Studies Depression Scale (CES-D) [41] to collect data on respondents’ depressive symptoms [42]. Respondents were asked how they felt during the past week through prompts such as “I felt depressed” and “I felt lonely," with possible responses on a scale of 0 (rarely/none of the time/1 day) to 3 (most/all of the time/5–7 days). The NLSY79 created a total CES-D score (ranging from 0 to 21) by summing the responses of all seven questions. A higher score indicates more depressive symptoms. The scale score was coded as missing if one item was missing. Compared to the original 20-item CES-D, this short form has similar or higher reliability and validity [43]. Prior studies have found a score of 8 or greater to have acceptable specificity and modest sensitivity with the standard CES-D cutoff score of 16 [43]; this study thus used this cutoff score to identify individuals with symptoms putting them at clinical risk of depression.

#### Work schedules

At every survey year, the NLSY79 asked participants about their work schedules. This study followed NLSY’s definitions and responses to create five work statuses. Specifically, a “standard” work schedule was defined as work beginning at 6 a.m. or later and ending by 6 p.m., “evenings” as work beginning at 2 p.m. or later and ending by midnight, “nights” as work beginning at 9 p.m. or later and ending by 8 a.m., and “variable” if the participant had either split or rotating shift or irregular hours. “Not working” was used when participants answered “not working at any job.” These five work statuses were used in the sequence analysis to arrive at possible clusters describing individuals’ employment patterns and trajectories.

#### Social position

This study used three indicators to define social position independent of employment patterns: gender, race-ethnicity, and education. The choice of these three indicators is to avoid reverse causality. For example, low-income status during adulthood tends to be highly associated with working nonstandard hours. However, nonstandard work schedules could lead to low-income status instead of vice versa. In this case, low-income status might be better conceptualized as a mediator instead of a moderator in the association between employment and health. The year 1979 was used as the data point to identify gender as either woman or man (as the reference group). In 1979, separate questions were asked about race and ethnicity. These two pieces of information were used to define four racial-ethnic groups: non-Hispanic White (reference group; Whites hereafter), non-Hispanic Black (Blacks hereafter), Hispanic, and others. Participants’ highest educational degree completed by age 23 was used to determine educational achievement with four dichotomous groups: less than a high school degree (<12 years of schooling), a high school degree (12 years of schooling, reference group), some college (13–15 years of schooling), and college or higher (16+ years of schooling).

#### Sociodemographic characteristics

A rich set of sociodemographic characteristics was considered in all analyses to address the potential unobserved heterogeneity between participants and selection bias that might explain the associations between employment patterns and sleep and health [1, 8]. These variables include age in 1979; background characteristics at age 14, including not living with both biological parents, parental education (i.e., less than high school, high school as the reference group, some college, or college or higher), and living location (suburban, rural, versus urban); region of residence at age 22 (Northeast, Midwest, West, versus South); any health issues that limited the ability to work by age 22; being a parent by age 22; ever experiencing poverty before age 23; ever receiving welfare before age 23; the number of years living in poverty between ages 22 and 49; the number of years receiving welfare between ages 22 and 49; number of marriages by age 49; number of children by age 49; average weekly working hours between ages 22 and 49; and occupations between ages 22 and 49. I defined poverty as family income at 100% of or under the federal poverty threshold. Welfare receipt was defined as receiving any assistance, including low-income cash transfers (e.g., AFDC or TANF), food assistance (e.g., food stamps or SNAP), or supplemental security income (SSI). Of note, to avoid reverse causality, I did not control for annual wages or income given the high correlation between these two variables and the type of work; instead, education, experiences with poverty and welfare receipt, and occupations were considered as proxies for resources available and accessible to respondents.

I created three dichotomous variables to measure average weekly working hours with a value of 1 and 0 otherwise to categorize participants as having (1) “mostly or only full-time hours” if they worked 35+ hours a week for at least half of the survey years (i.e., proportion of 0.50–0.99) between 22 and 49 (the reference group), (2) “mostly or only part-time hours” if they worked fewer than 35 hours a week for at least half of the survey years between 22 and 49, or (3) “mixed” if the participants worked about an equal share of survey years at full- and part-time hours between 22 and 49. Similarly, data on occupation were collected at each interview between ages 22 and 49. I created five occupational categories: mostly professional/managerial, mostly sales-related, mostly service-related (the reference group), mostly other occupations, and mixed. I used dichotomous variables to classify each participant’s primary occupation between 22 and 49. A person was considered mostly professional/managerial if they worked at least half the survey years between ages 22 and 49 in such an occupation. The “mixed” occupation category comprised participants who worked about an equal share of the survey years in at least two of the five occupation categories between 22 and 49.

#### Data analysis

Stata v.15 was used to perform the analyses in two steps. I first used sequence analysis to identify work schedule patterns between ages 22 and 49. I then conducted multiple regression analyses to examine the association between work schedule patterns (found in the sequence analysis) and the following health outcomes: sleep hours and quality, having poor health, SF-12 physical and mental functions, and having depressive symptoms.

When using a life-course perspective and focusing on the principles of lifespan development and timing, a sequence analysis is a well-suited statistical tool to chronologically classify the transitions between work schedule statuses over time [44]. To document the changes or transitions chronologically, this analysis used each year between the ages of 22 and 49 as the time axis, and the five work schedule statuses as the state or categorical variable tracked over time. I followed two steps to portray the work schedule trajectories over the working years (i.e., sequences) and then cluster the trajectories into groups. First, I calculated the similarity and dissimilarity between sequences using an optimal matching algorithm by setting the “costs” of turning one sequence into another [45, 46]. Following the sequence analysis literature, I set the insertion and deletion costs to be 1 and used the Needleman-Wunsch algorithm to calculate the substitution costs based on the transition rates between work schedule categories; when the transition is rare, the substitution cost is higher [47, 48]. I conducted additional sensitivity analyses using alternative theoretical-driven substitution (such as 2, 3, or other theoretically driven cost structures) to ensure the cluster solutions are not sensitive to cost-setting decisions [49]. The results affirm that they are not.

**Fig 1:**
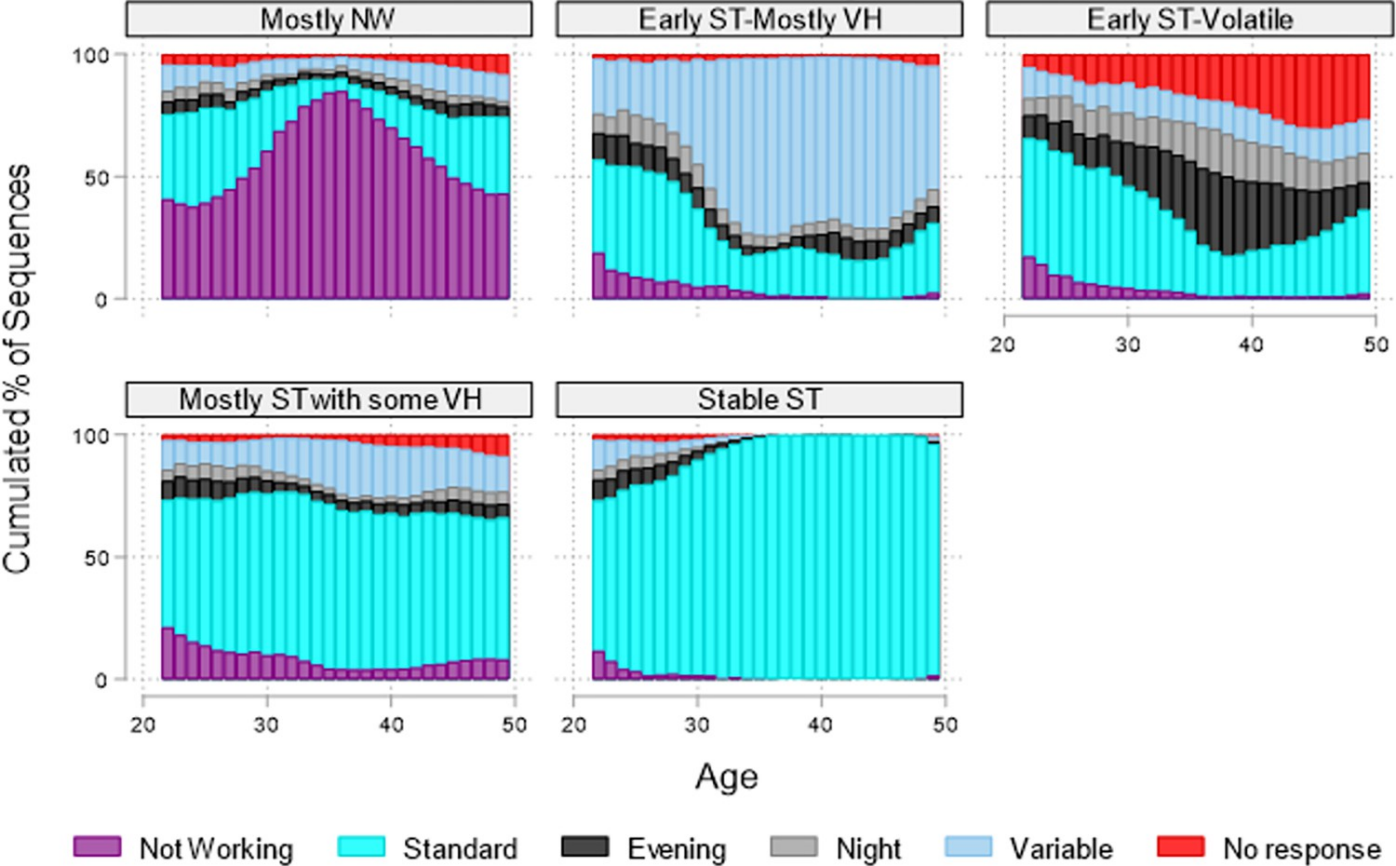
Distribution plot of five sequence clusters on work arrangements between ages 22 and 49, NLSY79.

The next step was to cluster similar sequences into a finite number of groups using Ward’s hierarchical fusion algorithm [45]. The stopping rules based on the Calinski and Harabasz pseudo-F index and the Duda-Hart index, as well as the conceptual meaning of clusters, were used to determine the ideal number of clusters [50]. Fig 1 presents the five sequence cluster solutions obtained, and S1 Table presents these diagnostic tests.

In the second step of the analysis, I used ordinary least squares (OLS) models to examine the associations between employment patterns and sleep hours and quality and SF-12 physical and mental functions, and I used logistic regression models to assess the associations between employment patterns and the likelihood of reporting poor health and having depressive symptoms (CES-D score > = 8). I then conducted post-estimations based on each multiple regression model to assess whether the regression estimates for the dependent variables were statistically significantly different between the five employment patterns. Next, I conducted interaction analyses to evaluate whether the associations between employment patterns and sleep and health might vary by social position. I conducted separate analyses by interacting the employment patterns with the following social position markers one at a time: race-ethnicity, gender, and education.

**Fig 2:**
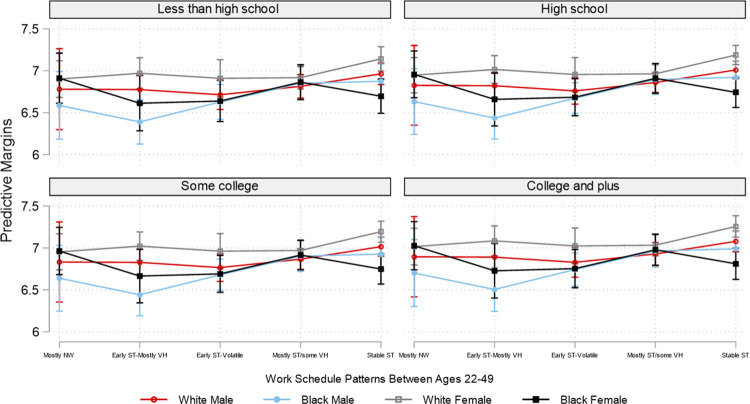
Adjusted predictions of sleep hours per day at age 50 by work schedule patterns, gender, race, and education. Note: ST: standard hours; VH: variable hours; NW: not working. The box plot displays the 95% confidence interval of each predicted estimate.

**Fig 3:**
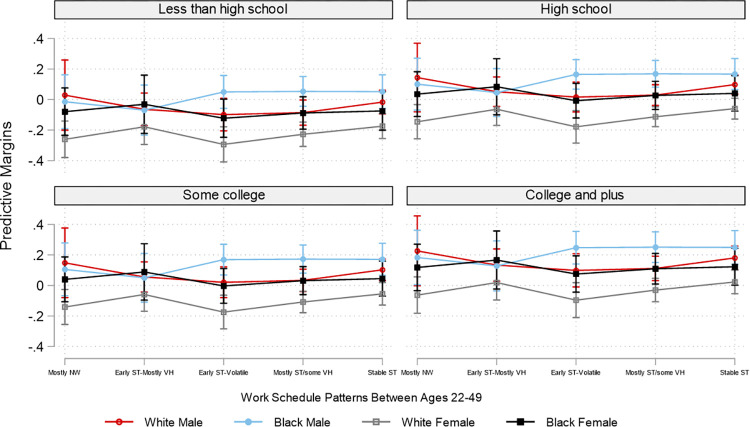
Adjusted predictions of sleep quality at age 50 by work schedule patterns, gender, race, and education. Note: ST: standard hours; VH: variable hours; NW: not working. The box plot displays the 95% confidence interval of each predicted estimate.

**Fig 4:**
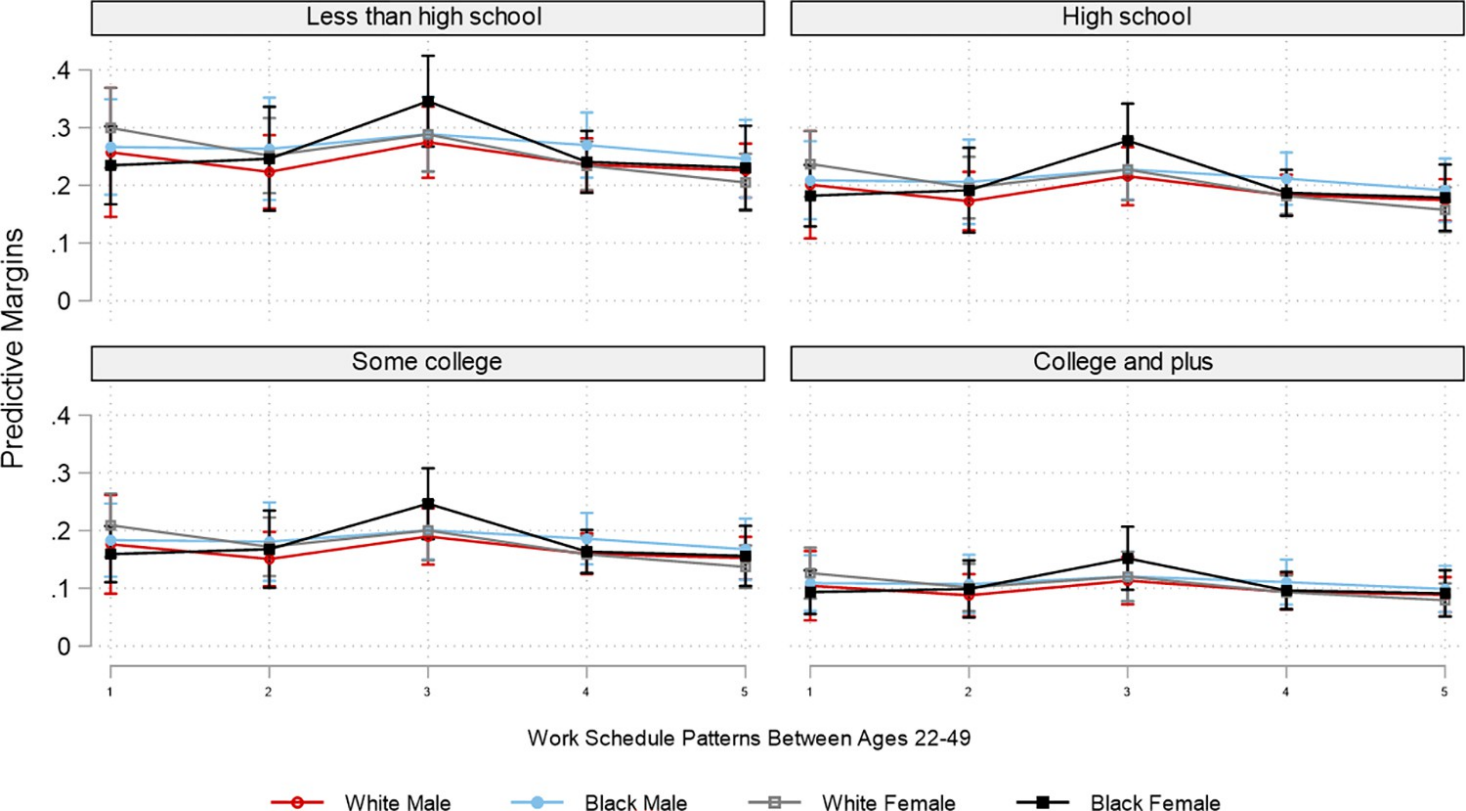
Adjusted predictions of poor health at age 50 by work schedule patterns, gender, race, and education. Note: ST: standard hours; VH: variable hours; NW: not working. The box plot displays the 95% confidence interval of each predicted estimate.

**Fig 5:**
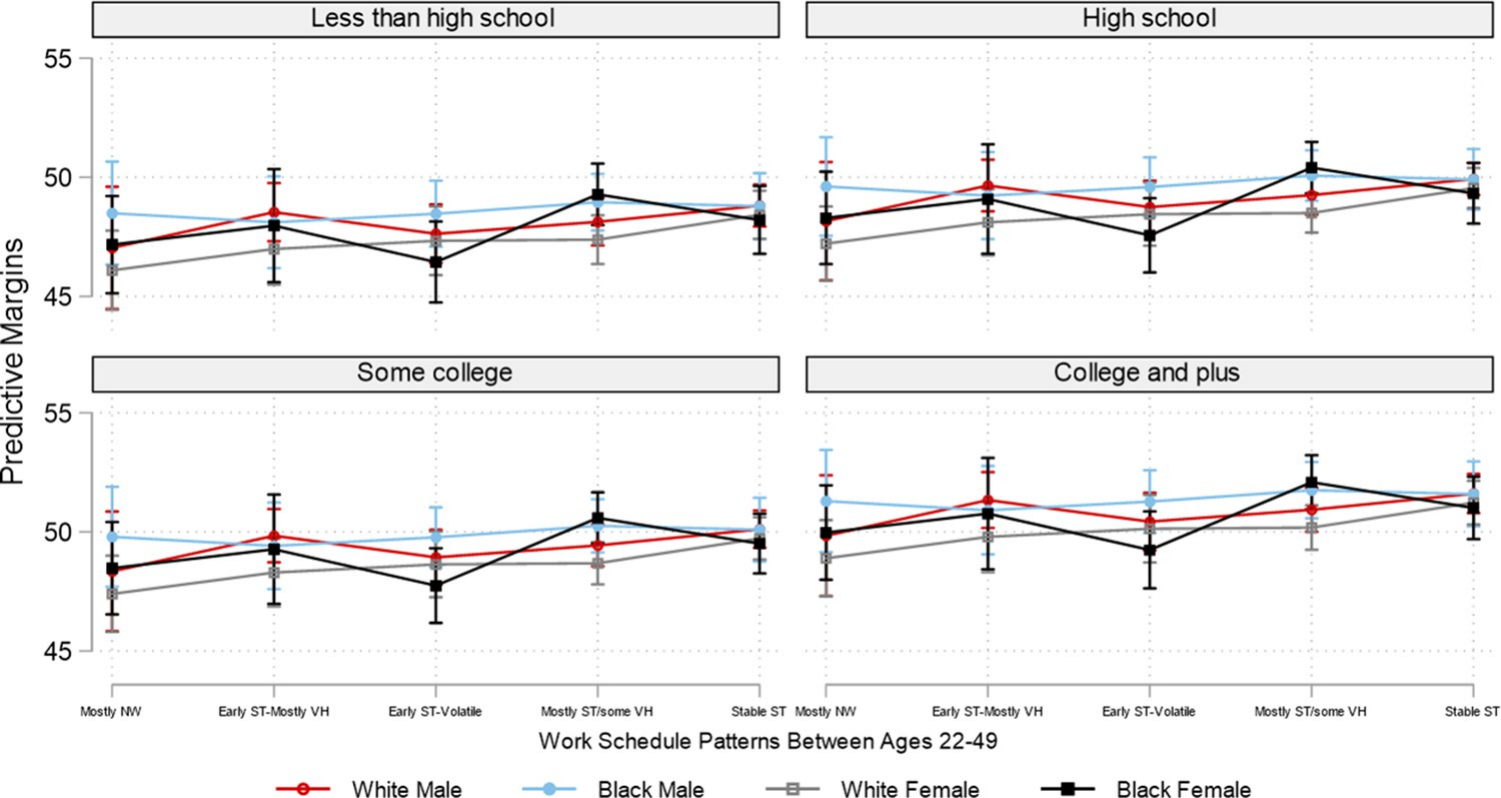
Adjusted predictions of SF-12 physical function at age 50 by work schedule patterns, gender, race, and education. Note: ST: standard hours; VH: variable hours; NW: not working. The box plot displays the 95% confidence interval of each predicted estimate.

**Fig 6:**
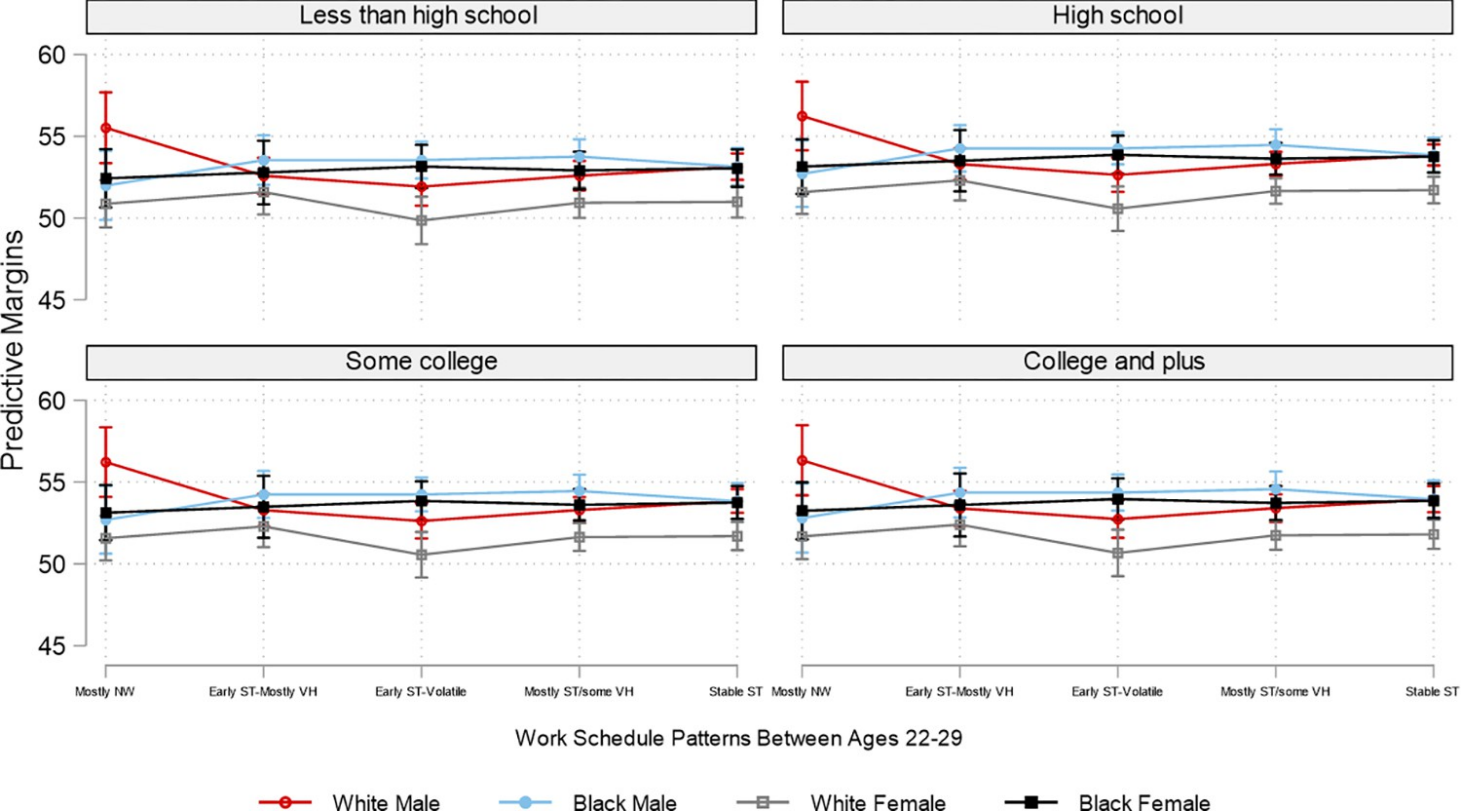
Adjusted predictions of SF-12 mental functions at age 50 by work schedule patterns, gender, race, and education. Note: ST: standard hours; VH: variable hours; NW: not working. The box plot displays the 95% confidence interval of each predicted estimate.

**Fig 7:**
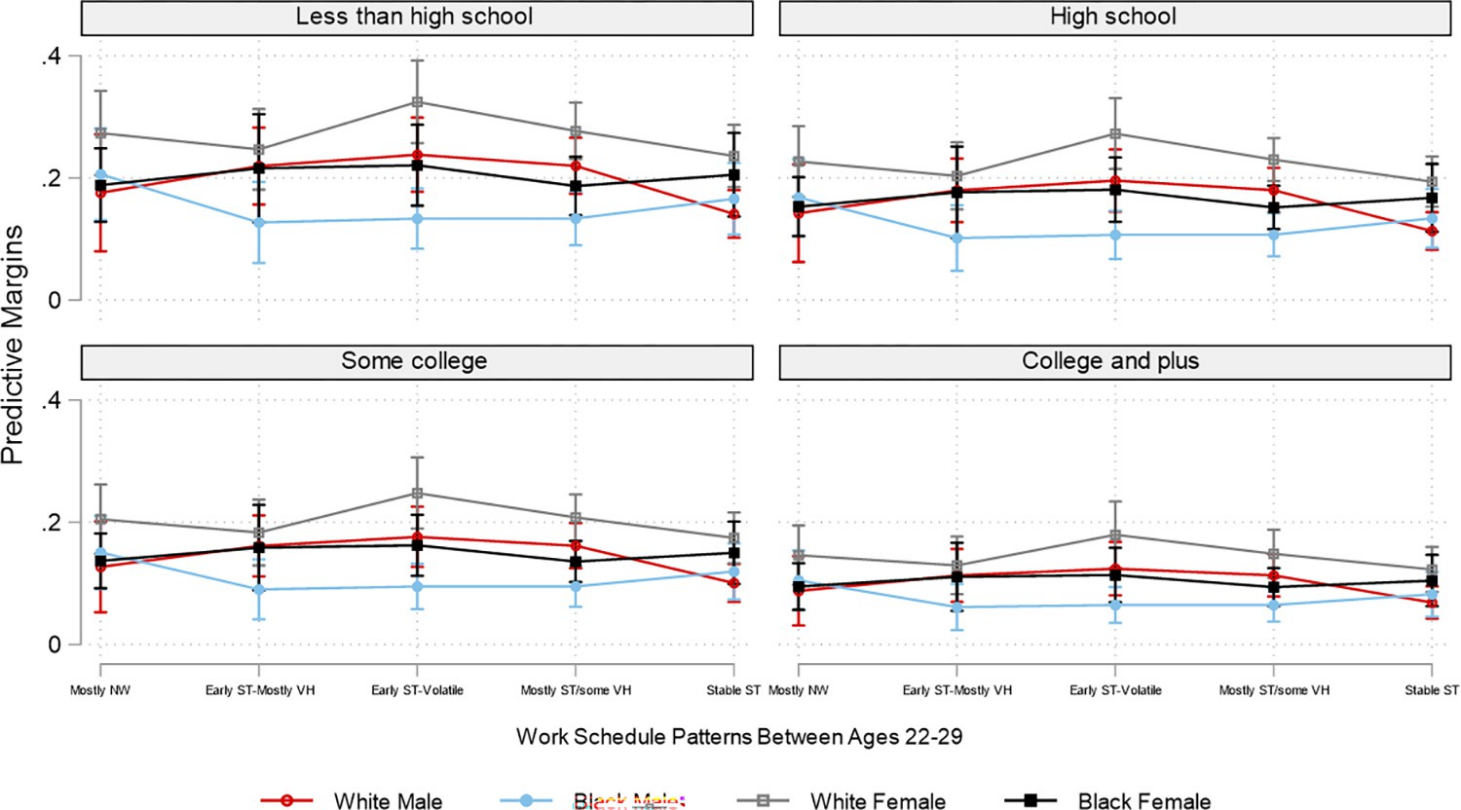
Adjusted predictions of depressive symptoms at age 50 by work schedule patterns, gender, race, and education. Note: ST: standard hours; VH: variable hours; NW: not working. The box plot displays the 95% confidence interval of each predicted estimate.

Due to the overwhelming number of combinations of employment patterns and the three social position markers, for brevity and for illustrative purposes, based on the interaction analyses, Figs 2–7 plot the predicted estimates of the number of sleep hours, sleep quality, poor health, SF-12 physical function, SF-12 mental function, and depressive symptoms against the work schedule patterns and the joint characteristics of race-ethnicity, gender, and education. The predicted probabilities in Figs 2–7 were produced by using the “margins” command in Stata based on the multivariate regression analyses. Of note, results for Hispanics were similar to but weaker than those comparing non-Hispanic Whites and non-Hispanic Blacks. Results for "Other" respondents were insignificant, primarily due to extremely small sample sizes. Therefore, the comparison between racial-ethnic groups in the Results section focuses on the Black–White differences.

## Results

### Descriptive picture

Table 1 displays all analyzed variables for the total sample (n = 7,336) and by employment cluster patterns between ages 22 and 49. Table 1 also presents the results of bivariate statistical tests to gauge differences between employment patterns in regard to sociodemographic characteristics. The focal independent variable in this analysis is employment patterns between ages 22 and 49. Fig 1 presents the distribution plot of the sequence analysis clusters of employment patterns between ages 22 and 49. About 60% of the NLSY79 participants had employment patterns involving mostly standard hours (ST) throughout their working years: 35% worked "mostly ST with some variable hours (VH)," and 26% worked "stable ST." A decent share of participants (17%) had an employment pattern characterized as working standard hours early in their careers (20s) but transitioning into a variety of work schedules (during their early 30s). This group is labeled "early ST-volatile." Another 12% of respondents had a similar employment pattern of working standard hours during their early working years but switched into mainly variable hours (labeled "early ST-mostly VH"). Finally, About 11% of respondents had an employment pattern characterized as "mostly not working (NW)."

**Table 1:** Descriptive statistics for analyzed variables between ages 22 and 49, NLSY79 (N = 7,336).

		By work schedule patterns between ages 22–48/49					Sig.
	Total	Mostly NW (n = 788, 10.74%)	Early ST-Mostly VH (n = 845, 11.52%)	Early ST-Volatile (n = 1,255, 17.11%)	Mostly ST with some VH (n = 2,553, 34.80%)	Stable ST (n = 1,895, 25.83%)	
Age	17.57 (2.23)	17.67 (2.24)	17.49 (2.25)	17.47 (2.17)	17.52 (2.26)	17.70 (2.21)	*
Gender (%)							
Female	52.18	72.72	45.92	44.54	55.62	46.86	***
Race-ethnicity (%)							***
Non-Hispanic White	50.27	38.83	57.16	41.99	48.92	57.94	
Non-Hispanic Black	29.72	39.21	27.81	38.01	28.63	21.90	
Hispanic	19.45	21.70	14.32	18.80	21.35	18.68	
Others	1.08	0.25	0.71	1.20	1.10	1.48	
Immigrant status (%)							**
First generation	5.28	6.85	3.43	6.06	4.90	5.44	
Second generation	8.86	8.88	8.88	8.37	8.93	9.08	
Third+ generation	85.86	84.26	87.69	85.58	86.17	85.49	
Background at age 14							
Parental education (%)							***
Less than high school	34.49	45.05	26.04	38.41	36.66	28.34	
High school	38.55	33.76	41.18	38.73	37.68	40.42	
Some college	11.78	10.03	13.49	10.60	10.89	13.72	
College and plus	15.18	11.17	19.29	12.27	14.77	17.52	
Not living with both biological parents (%)	31.22	41.75	31.24	36.25	30.83	24.01	***
Residence (%)							*
Urban	79.17	83.38	77.25	81.27	79.21	77.84	
Suburban	15.72	13.32	17.06	14.81	15.91	16.66	
Rural	4.78	3.30	5.69	3.92	4.88	5.50	
Any health issues limiting work by age 22 (%)	9.68	16.50	10.18	9.40	9.75	6.70	***
Education by age 23	12.54 (2.17)	11.66 (2.27)	12.88 (1.95)	12.26 (1.94)	12.48 (2.19)	13.04 (2.20)	***
Less than high school (%)	19.68	36.80	14.56	20.00	20.56	13.46	***
High school (%)	42.91	39.09	42.60	49.48	42.34	41.06	
Some college (%)	23.38	16.88	27.69	22.47	24.05	23.85	
College and plus (%)	14.03	7.23	15.15	8.05	13.04	21.64	
Residence at age 22 (%)							
Rural (vs. urban)	17.88	14.85	19.29	17.29	18.45	18.15	
Region of residence at age 22 (%)							**
Northeast	18.02	20.69	17.16	20.96	15.90	18.21	
Midwest	24.00	21.32	24.97	24.78	23.97	24.22	
South	37.88	36.80	37.28	37.05	38.31	38.58	
West	20.09	21.19	20.59	17.21	21.82	19.00	
Marital status at age 22 (%)							***
Married	28.16	28.30	26.63	26.85	30.40	26.65	
Previously married	5.41	8.38	6.27	5.58	5.29	3.85	
Never married	66.42	63.32	67.10	67.57	64.32	69.50	
Being a parent by age 22 (%)	29.05	42.26	24.73	28.84	32.24	21.32	***
Ever receiving welfare before age 22 (%)	21.37	38.71	19.64	21.27	23.31	12.40	***
Ever been in poverty before age 22 (%)	43.55	60.79	40.00	45.66	44.58	35.20	***
Number of marriages by age 48/49	1.26 (1.28)	1.11 (1.29)	1.36 (1.30)	1.17 (1.32)	1.33 (1.32)	1.24 (1.32)	***
Number of children by age 48/49	1.96 (1.36)	2.43 (1.62)	1.85 (1.32)	1.84 (1.44)	1.98 (1.31)	1.88 (1.24)	***
Number of years receiving welfare between ages 22 and 48/49	2.35 (4.38)	6.67 (7.01)	1.82 (3.45)	2.00 (3.65)	2.41 (4.21)	0.92 (2.40)	***
Number of years living in poverty between ages 22-48/49	2.79 (3.98)	6.96 (6.00)	2.07 (2.92)	2.49 (3.30)	2.96 (3.89)	1.34 (2.32)	***
Weekly work hours between ages 22-48/49 (%)							***
Mostly full-time (> = 35 hrs/wk)	76.31	13.07	79.53	82.87	77.63	95.04	
Mostly part-time (1–34 hrs/wk)	21.40	82.87	18.70	14.18	19.51	4.38	
Mixed	2.29	4.06	1.78	2.95	2.86	0.58	
Occupation between ages 22-48/49 (%)							***
Mostly professional/managerial	24.47	11.55	28.88	16.41	22.37	36.04	
Mostly sales-related	2.59	3.55	2.01	1.59	2.78	2.85	
Mostly service-related	36.68	51.78	34.20	41.91	37.56	26.86	
Mostly in other occupation	30.29	21.45	27.34	34.26	31.41	31.13	
Mixed	5.97	11.68	7.57	5.82	5.88	3.11	
Health Outcomes at Age 50							
Sleep hours on a weekday (n = 7,320)	6.62 (1.41)	6.57 (1.77)	6.54 (1.34)	6.45 (1.45)	6.64 (1.41)	6.76 (1.22)	***
Sleep hours on a weekend day (n = 7,314)	7.21 (1.64)	7.03 (1.98)	7.09 (1.51)	7.08 (1.75)	7.23 (1.66)	7.42 (1.39)	***
Sleep hours per day over a week (n = 7,324)	6.92 (1.39)	6.79 (1.75)	6.82 (1.32)	6.76 (1.47)	6.93 (1.40)	7.08 (1.16)	***
Sleep quality (n = 7,336)	0.01 (0.81)	-0.23 (0.93)	0.04 (0.82)	-0.00 (0.82)	0.00 (0.81)	0.14 (0.73)	***
Poor health (%) (n = 7,334)	19.59	34.26	15.98	23.21	19.65	12.61	***
SF-12 Physical function (n = 7,262)	49.28 (10.14)	44.51 (12.74)	49.60 (10.42)	48.75 (10.16)	49.29 (9.97)	51.43 (8.15)	***
SF-12 Mental function (n = 7,262)	52.96 (8.81)	49.94 (10.86)	53.37 (8.34)	52.84 (9.11)	52.95 (8.90)	54.11 (7.34)	***
CES-D (> = 8) (%) (n = 7,271)	16.70	27.98	15.54	17.62	17.21	11.23	***

*Note*. NW: not working; ST: standard hours; VH: variable hours. Standard deviations are in parentheses. Chi-square (for two categorical variables), T-test, or ANOVA were used to determine statistical significance between groups

* *p* < .05

** *p* < .01

*** *p* < .001.

Table 1 also shows sociodemographic characteristics of the sample. NLSY79 contains slightly more women than men. Approximately half of the participants were non-Hispanic White, with another third identified as non-Hispanic Black, 19% as Hispanic, and about 1% as some other racial group. The majority were U.S.-born. In addition, at age 14, more than 70% of the NLSY79 respondents had parents with a high school degree or less, one-third were not living with both biological parents, and almost 80% lived in urban areas. Nearly 10% of these young adults reported health issues that limited work capacity by age 22. By age 23, for about 20% of these young adults, less than high school was their highest educational attainment, 43% had a high school degree, 23% had some college, and 14% had a college or above education. Roughly 30% of the participants were married at age 22, and about 29% had become parents by age 22. Finally, before age 23, about 44% of the participants had experienced poverty, and about 21% had received welfare assistance.

Furthermore, between ages 22 and 49, participants experienced an average of one marriage and had an average of two children. In addition, participants spent an average of two to three years experiencing poverty and received welfare assistance for an average of over two years. More than three-fourths of the participants mainly worked full-time (i.e., 35 or more hours per week). About a quarter of the participants mostly worked in professional/managerial occupations, more than one-third had primarily service-related occupations, and another 30% had jobs primarily in occupations other than professional/managerial, sales-, or service-related.

Regarding the outcome variables considered in this analysis, the average number of sleep hours per day on a weekday was 6.62 (SD = 1.41) and 7.21 (SD = 1.64) on the weekend; combined, across a 7-day week, participants slept an average of 6.92 hours (SD = 1.39). The average sleep quality of the analyzed sample was at the mean value. About 20% of the participants reported their general health status was either fair or poor. The average SF-12 physical function was 49.28 (SD = 10.14), slightly below the national average, and the average SF-12 mental function was 52.96 (SD = 8.81), slightly above the national average. These findings are consistent with the NLSY79’s reported statistics [34]. Approximately 17% of the respondents reported having depressive symptoms (CES-D scores > = 8) at age 50.

The bivariate statistical analyses shown in Table 1 suggest that people with employment patterns of “stable ST” had comparatively advantaged characteristics in terms of being non-Hispanic White, having a college or above education by age 23, being less likely to have been exposed to poverty or welfare assistance by age 22, and having a lower-than-average percentage of health issues limiting their ability to work. The next groups with somewhat advantaged sociodemographic characteristics were participants with an employment pattern of either “early ST-mostly VH” or “mostly ST with some VH;” the notable differences between these two groups were the former being more likely to be a man and non-Hispanic White and the latter being more likely to be a female and Hispanic. In contrast, being a man, being non-Hispanic Black, and having a high school degree were more likely to be associated with the “early ST-volatile” employment pattern. Of importance, participants with an employment pattern of “mostly NW” tended to have somewhat disadvantaged sociodemographic backgrounds. For instance, they were likely to be either non-Hispanic Black or Hispanic, to have less than a high school education, to have health issues limiting work, to have become parents by age 22, to have been exposed to poverty or welfare by age 22, and to experience more years of poverty and welfare assistance after age 22. Given these differences in sociodemographic backgrounds for the employment patterns, it is not surprising to find that, generally, people with the “stable ST” employment pattern between ages 22 and 49 had the most favorable sleep and health outcomes, and that people with the “mostly NW” employment pattern had the worst sleep and health outcomes. Those with the “early ST-volatile” employment pattern had the second-worst sleep and health outcomes.

### Multiple regression estimates of work schedule patterns on sleep and health

Tables 2 and 3 report multiple regression estimates of employment patterns on sleep and health outcomes, with Table 2 reporting the hours and quality of sleep (OLS regression) along with the likelihood of self-reporting poor health (logistic regression) and Table 3 reporting SF-12 physical and mental functions (OLS regression) along with the likelihood of self-reporting depressive symptoms (logistic regression). All sociodemographic characteristics detailed in the Measures section were considered in all analyses. On the whole, results in Tables 2 and 3 indicate that employment patterns matter to sleep and health. Specifically, compared to the pattern of mostly stable standard hours (“stable ST”), having an employment pattern of working standard hours during early career years (age 20s) but transitioning into volatile schedules after age 30 (“early ST-volatile”) was statistically significantly associated with fewer hours of sleep per day, lower quality of sleep, a higher likelihood of self-reporting poor health at age 50, lower scores on SF-12 physical and mental functions, and a higher likelihood of having depressive symptoms. Compared to the “stable ST” pattern, people with an employment pattern of working mostly standard hours but with some variable hours (“mostly ST with some VH”) also had significantly worse sleep and health outcomes, except for a nonsignificant effect on SF-12 mental function. People with an employment pattern of having standard hours during their 20s but transitioning into mostly variable hours after age 30 (“early ST-mostly VH”) had significantly fewer hours of sleep per day and significantly lower SF-12 physical function scores than those with the “stable ST” pattern. Lastly, people with an employment pattern of mostly not working (“mostly NW”) reported a significantly higher likelihood of poor health and significantly lower SF-12 physical function than those with the “stable ST” pattern. In addition, post-estimation Wald test results (not shown, available upon request) indicate that individuals with the “early ST-volatile” employment pattern (1) slept significantly fewer hours (b = -0.24 vs. b = -0.10, χ^2^ = 7.42, *p* < .01), reported significantly lower SF-12 physical function (b = -1.42 vs. b = -0.62, χ^2^ = 5.53, *p* < .05), and were more likely to report poor health (b = 0.45 vs. b = 0.18, χ^2^ = 9.08, *p* < .01) than those with the “mostly ST with some VH” pattern and (2) were more likely to report poor health than those with the “early ST-mostly VH” pattern (b = 0.45 vs. b = 0.08, χ^2^ = 9.39, *p* < .01). Furthermore, individuals with the employment pattern of “early ST-mostly VH” had significantly fewer hours of sleep per day during the week compared to those with the “mostly ST with some VH” pattern (b = -0.23 vs. b = -0.10, χ^2^ = 6.28, *p* < .01). The post-estimation Wald tests detected no other statistically significant differences among employment patterns.

**Table 2:** Multiple regression estimates of work schedules between ages 22 and 48/49 on health outcomes at age 50.

	Hours of sleep per day/wk	Sleep quality	Poor health
	b (se) [95% CI]	b (se) [95% CI]	b (se) [95% CI]
Work schedules (vs. stable ST)			
Mostly NW	-0.14 (0.08) [-0.30, 0.01]	-0.07 (0.04) [-0.16, 0.01]	0.27 (0.14) [0.00, 0.54] *
Early ST-mostly VH	-0.23 (0.05) [-0.33, -0.13] ***	-0.05 (0.03) [-0.11, 0.02]	0.08 (0.12) [-0.16, 0.32]
Early ST-volatile	-0.24 (0.05) [-0.34, -0.14] ***	-0.08 (0.03) [-0.13, -0.02] **	0.45 (0.10) [0.25, 0.65] ***
Mostly ST with some VH	-0.10 (0.04) [-0.17, -0.02] *	-0.05 (0.02) [-0.09, -0.00] *	0.18 (0.09) [0.00, 0.36] *
Age	0.02 (0.01) [0.00, 0.04] **	0.00 (0.00) [-0.00, 0.01]	0.02 (0.02) [-0.01, 0.05]
Female	0.09 (0.04) [0.02, 0.17] *	-0.14 (0.02) [-0.18, -0.09] ***	-0.02 (0.08) [-0.18, 0.14]
Race-ethnicity (vs. Non-Hispanic White)			
Non-Hispanic Black	-0.15 (0.05) [-0.24, -0.06]***	0.12 (0.02) [0.07, 0.17] ***	0.08 (0.09) [-0.09, 0.25]
Hispanic	0.03 (0.05) [-0.08, 0.13]	0.10 (0.03) [0.04, 0.16] ***	0.03 (0.10) [-0.17, 0.23]
Others	0.01 (0.13) [-0.25, 0.28]	0.10 (0.08) [-0.06, 0.26]	0.08 (0.30) [-0.51, 0.68]
Family background at age 14			
Parental education (vs. High school)			
Less than high school	-0.06 (0.04) [-0.15, 0.02]	-0.05 (0.02) [-0.10, -0.01] *	0.08 (0.08) [-0.07, 0.24]
Some college	-0.03 (0.05) [-0.14, 0.07]	-0.04 (0.03) [-0.11, 0.01]	-0.01 (0.11) [-0.23, 0.21]
College and plus	0.04 (0.05) [-0.05, 0.14]	-0.01 (0.03) [-0.06, 0.04]	-0.00 (0.12) [-0.23, 0.22]
Not living with both bio parents	0.01 (0.04) [-0.06, 0.09]	-0.00 (0.02) [-0.05, 0.04]	-0.12 (0.07) [-0.26, 0.02]
Residence (vs. urban)			
Suburban	-0.07 (0.04) [-0.15, 0.02]	0.03 (0.03) [-0.02, 0.08]	-0.03 (0.09) [-0.20, 0.15]
Rural	-0.03 (0.08) [-0.18, 0.12]	0.01 (0.04) [-0.07, 0.09]	-0.03 (0.16) [-0.34, 0.27]
Immigrant status (vs. third+ generation)			
First generation	0.09 (0.08) [-0.06, 0.24]	0.03 (0.05) [-0.06, 0.12]	-0.14 (0.16) [-0.46, 0.18]
Second generation	-0.00 (0.06) [-0.11, 0.11]	0.01 (0.03) [-0.06, 0.08]	-0.05 (0.12) [-0.28, 0.18]
Education by age 23 (vs. High school)			
Less than high school	-0.04 (0.06) [-0.15, 0.06]	-0.12 (0.03) [-0.18, -0.06] ***	0.36 (0.08) [0.20, 0.52] ***
Some college	0.00 (0.04) [-0.08, 0.08]	0.00 (0.02) [-0.04, 0.05]	-0.17 (0.09) [-0.35, -0.00] *
College and plus	0.07 (0.05) [-0.04, 0.17]	0.08 (0.03) [0.02, 0.15] **	-0.81 (0.16) [-1.14, -0.49] ***
Marital status at age 22 (vs. Married)			
Previously married	-0.16 (0.09) [-0.33, 0.01]	-0.10 (0.05) [-0.20, -0.01] *	0.04 (0.14) [-0.23, 0.31]
Never married	-0.07 (0.04) [-0.16, 0.01]	-0.00 (0.02) [-0.05, 0.05]	-0.09 (0.08) [-0.26, 0.07]
Becoming a parent by age 22	-0.09 (0.05) [-0.19, 0.02]	-0.03 (0.03) [-0.09, 0.02]	-0.10 (0.09) [-0.28, 0.08]
Ever in poverty before age 22	-0.00 (0.04) [-0.08, 0.08]	0.01 (0.02) [-0.03, 0.06]	-0.04 (0.08) [-0.20, 0.11]
Ever receiving welfare before age 22	-0.00 (0.06) [-0.12, 0.12]	0.04 (0.03) [-0.02, 0.11]	-0.16 (0.10) [-0.36, 0.05]
Any health issue limiting work by age 22	-0.10 (0.06) [-0.22, 0.02]	-0.13 (0.03) [-0.20, -0.07] ***	0.53 (0.10) [0.34, 0.71] ***
Region of residence at age 22 (vs. South)			
Northeast	-0.12 (0.05) [-0.21, -0.03] *	-0.03 (0.03) [-0.08, 0.02]	-0.09 (0.09) [-0.28, 0.09]
Midwest	0.06 (0.04) [-0.02, 0.15]	0.02 (0.02) [-0.03, 0.07]	-0.15 (0.09) [-0.33, 0.02]
West	0.06 (0.05) [-0.03, 0.15]	0.05 (0.03) [-0.01, 0.10]	-0.06 (0.09) [-0.24, 0.12]
Characteristics between ages 22 and 48/49			
# of marriages	-0.04 (0.01) [-0.06, -0.01] **	-0.02 (0.01) [-0.03, -0.00] *	0.04 (0.02) [-0.01, 0.09]
# of children	-0.02 (0.01) [-0.05, 0.01]	0.02 (0.01) [0.01, 0.04] **	-0.09 (0.02) [-0.14, -0.04] ***
# of years receiving welfare	-0.01 (0.01) [-0.03, 0.00]	-0.02 (0.00) [-0.03, -0.01] ***	0.08 (0.01) [0.06, 0.10] ***
# of years in poverty	-0.00 (0.01) [-0.02, 0.01]	-0.01 (0.00) [-0.02, -0.00] *	0.04 (0.01) [0.01, 0.06] **
Average weekly work hours (vs. only or mostly full-time)			
Only or mostly part-time (<35 hrs/wk)	0.03 (0.06) [-0.08, 0.14]	-0.05 (0.03) [-0.12, -0.01]	0.16 (0.10) [-0.03, 0.35]
Mixed	-0.11 (0.12) [-0.36, 0.13]	-0.15 (0.08) [-0.31, -0.01] *	0.40 (0.18) [0.04, 0.76] *
Occupations (vs. mostly service occupation)			
Mostly professional/managerial	0.04 (0.05) [-0.05, 0.13]	0.05 (0.03) [0.00, 0.11] *	-0.34 (0.11) [-0.56, -0.12] **
Mostly sales	-0.05 (0.09) [-0.23, 0.14]	0.07 (0.06) [-0.05, 0.18]	-0.23 (0.23) [-0.68, 0.22]
Mostly other occupations	-0.02 (0.05) [-0.12, 0.08]	0.00 (0.03) [-0.05, 0.06]	0.11 (0.09) [-0.06, 0.29]
Mixed	-0.01 (0.08) [-0.16, 0.14]	0.05 (0.04) [-0.03, 0.14]	0.16 (0.13) [-0.09, 0.41]
Constant	6.89 (0.16) [6.58, 7.20] ***	0.07 (0.09) [-0.11, 0.24]	-2.01 (0.31) [-2.63, -1.41] ***
R-square / Pseudo R-square	.029	.075	.102
N	7,324	7,336	7,334

*Note*. Ordinary least squares regressions were used for sleep hours and quality, and a logistic regression was used for poor health outcomes. Numbers represent unstandardized coefficients with robust-adjusted standard errors in parentheses and a 95% confidence interval in the brackets

* *p* < .05

** *p* < .01

*** *p* < .001.

**Table 3:** Multiple regression estimates of work schedules between ages 22 and 48/49 on health outcomes at age 50.

	SF-12 Physical Function	SF-12 Mental Function	CES-D (> = 8)
	b (se) [95% CI]	b (se) [95% CI]	b (se) [95% CI]
Work schedules (vs. stable ST)			
Mostly NW	-1.97 (0.56) [-3.07, -0.86] ***	-0.65 (0.49) [-1.60, 0.30]	0.26 (0.14) [-0.01, 0.54]
Early ST-mostly VH	-0.89 (0.39) [-1.66, -0.13] *	-0.20 (0.34) [-0.86, 0.45]	0.22 (0.12) [-0.03, 0.46]
Early ST-volatile	-1.42 (0.34) [-2.10, -0.75] ***	-0.82 (0.31) [-1.43, -0.21] **	0.36 (0.11) [0.14, 0.58] ***
Mostly ST with some VH	-0.62 (0.27) [-1.14. -0.09] *	-0.25 (0.25) [-0.74, 0.24]	0.22 (0.10) [0.03, 0.40] *
Age	-0.01 (0.05) [-0.11, 0.10]	-0.12 (0.04) [-0.21, -0.03]**	0.03 (0.02) [0.00, 0.06] *
Female	-0.52 (0.27) [-1.06, 0.01]	-1.24 (0.24) [-1.73, -0.76] ***	0.30 (0.09) [0.12, 0.47] ***
Race-ethnicity (vs. Non-Hispanic White)			
Non-Hispanic Black	0.64 (0.32) [0.02, 1.26] *	1.40 (0.28) [0.86, 1.95] ***	-0.42 (0.09) [-0.60, -0.24] ***
Hispanic	0.52 (0.38) [-0.22, 1.27]	1.05 (0.34) [0.40, 1.72] **	-0.31 (0.11) [-0.53, -0.09] **
Others	1.08 (0.92) [-0.72, 2.88]	0.22 (1.07) [-1.87, 2.32]	-0.35 (0.36) [-1.05, 0.35]
Family background at age 14			
Parental education (vs. High school)			
Less than high school	-1.02 (0.30) [-1.61, -0.44] ***	0.20 (0.26) [-0.32, 0.72]	-0.02 (0.08) [-0.18 0.15]
Some college	-0.36 (0.37) [-1.09, 0.36]	-0.36 (0.34) [-1.03, 0.30]	0.10 (0.11) [-0.12, 0.32]
College and plus	-0.14 (0.33) [-0.79, 0.51]	-0.72 (0.31) [-1.33, -0.12] *	0.11 (0.11) [-0.11, 0.34]
Not living with both bio parents	0.22 (0.27) [-0.31, 0.75]	0.11 (0.24) [-0.35, 0.58]	0.01 (0.08) [-0.14, 0.15]
Residence (vs. urban)			
Suburban	-0.06 (0.32) [-0.68, 0.57]	0.55 (0.27) [0.02. 1.07] *	-0.07 (0.09) [-0.25, 0.11]
Rural	-0.26 (0.53) [-1.30, 0.77]	0.64 (0.45) [-0.24, 1.53]	-0.06 (0.16) [-0.38, 0.26]
Immigrant status (vs. third+ generation)			
First generation	1.58 (0.53) [0.54, 2.62] **	-0.69 (0.52) [-1.71, 0.32]	-0.19 (0.18) [-0.54, 0.16]
Second generation	0.65 (0.41) [-0.15, 1.46]	0.05 (0.38) [-0.70, 0.79]	-0.07 (0.13) [-0.32, 0.17]
Education by age 23 (vs. High school)			
Less than high school	-1.20 (0.38) [-1.95, -0.45] **	-0.77 (0.34) [-1.43, -0.11] *	0.26 (0.09) [0.08, 0.44] **
Some college	0.18 (0.30) [-0.41, 0.77]	0.03 (0.27) [-0.49, 0.55]	-0.13 (0.09) [-0.32, 0.05]
College and plus	1.68 (0.36) [0.98, 2.39] ***	0.13 (0.34) [-0.53, 0.79]	-0.57 (0.15) [-0.86, -0.28] ***
Marital status at age 22 (vs. Married)			
Previously married	-0.41 (0.62) [-1.62, 0.80]	-1.24 (0.55) [-2.32, -0.15] *	0.11 (0.14) [-0.16, 0.38]
Never married	0.47 (0.31) [-0.14, 1.08]	0.05 (0.27) [-0.48, 0.58]	-0.03 (0.09) [-0.20, 0.14]
Becoming a parent by age 22	0.21 (0.36) [-0.50, 0.92]	0.20 (0.32) [-0.42, 0.82]	0.12 (0.10) [-0.07, 0.31]
Ever in poverty before age 22	0.41 (0.28) [-0.13, 0.95]	0.39 (0.25) [[-0.10, 0.89]	-0.02 (0.08) [-0.19, 0.14]
Ever receiving welfare before age 22	1.14 (0.42) [0.31, 1.96] **	0.02 (0.38) [-0.72, 0.76]	-0.25 (0.11) [-0.47, -0.03] *
Any health issue limiting work by age 22	-3.19 (0.46) [-4.09, -2.29] ***	-0.90 (0.39) [-1.67, -0.14]*	0.37 (0.10) [0.17, 0.56] ***
Region of residence at age 22 (vs. South)			
Northeast	0.70 (0.32) [0.07, 1.34] *	-0.35 (0.31) [-0.95, 0.25]	0.04 (0.10) [-0.15, 0.23]
Midwest	0.97 (0.30) [0.37, 1.57] ***	0.31 (0.28) [-0.22, 0.85]	-0.19 (0.09) [-0.37, -0.01] *
West	0.32 (0.34) [-0.34, 0.98]	0.38 (0.30) [-0.20, 0.97]	-0.01 (0.10) [-0.20, 0.18]
Characteristics between ages 22 and 48/49			
# of marriages	-0.17 (0.09) [-0.35, 0.02]	-0.31 (0.08) [-0.48, -0.15] ***	0.11 (0.02) [0.06, 0.16] ***
# of children	0.45 (0.09) [0.27, 0.64] ***	0.33 (0.08) [0.17, 0.49] ***	-0.11 (0.03) [-0.16, -0.05] ***
# of years receiving welfare	-0.55 (0.06) [-0.66, -0.44] ***	-0.17 (0.05) [-0.27, -0.07] ***	0.05 (0.01) [0.03, 0.08] ***
# of years in poverty	-0.10 (0.06) [-0.22, 0.02]	-0.28 (0.05) [-0.38, -0.17] ***	0.06 (0.01) [0.03, 0.08] ***
Average weekly work hours (vs. only or mostly full-time)			
Only or mostly part-time (<35 hrs/wk)	-0.82 (0.39) [-1.58, -0.05] *	-1.21 (0.35) [-1.90, -0.53] ***	0.16 (0.10) [-0.03, 0.35]
Mixed	-1.78 (0.94) [-3.62, 0.06]	-0.97 (0.78) [-2.50, 0.57]	0.22 (0.21) [-0.18, 0.63]
Occupations (vs. mostly service occupation)			
Mostly professional/managerial	1.04 (0.32) [0.41, 1.67] ***	0.45 (0.29) [-0.11, 1.02]	-0.19 (0.11) [-0.41, 0.02]
Mostly sales	1.51 (0.66) [0.22, 2.81] *	-0.07 (0.61) [-1.28, 1.14]	-0.15 (0.22) [-0.59, 0.29]
Mostly other occupations	-0.38 (0.35) [-1.06, 0.30]	-0.27 (0.30) [-0.87, 0.33]	0.01 (0.10) [-0.18, 0.20]
Mixed	-0.74 (0.57) [-1.85, 0.37]	0.19 (0.45) [-0.69, 1.07]	-0.25 (0.14) [-0.53, 0.04]
Constant	50.38 (1.07) [48.29, 52.48] ***	56.45 (0.93) [54.62, 58.28] ***	-2.58 (0.32) [-3.20, -1.96] ***
R-square / Pseudo R-square	.139	.073	.074
N	7,262	7,262	7,271

*Note*. Ordinary least squares regressions were used for SF-12 physical and mental functions, and a logistic regression was used for CES-D outcomes. Numbers represent unstandardized coefficients with robust-adjusted standard errors in parentheses and a 95% confidence interval in the brackets

* *p* < .05

** *p* < .01

*** *p* < .001.

As expected, age, gender, race-ethnicity, education, marital status, health issues limiting work capacity, years of receiving welfare or living in poverty, number of marriages and children, weekly working hours, and occupations were by and large significantly associated with hours and quality of sleep and other health outcomes. However, gender and race-ethnicity did not make a difference in the likelihood of self-reporting poor health. Specifically, people occupying vulnerable social positions (e.g., women, less than a high school education, previously married, having health limitations, multiple marriages, more experiences of poverty and welfare, not having full-time work status) tended to report lower sleep quality and lower SF-12 physical and mental functions, and were more likely to self-report poor health and having depressive symptoms. Note that, compared to men, women reported significantly more hours of sleep but substantially lower sleep quality. Moreover, compared to non-Hispanic White peers, non-Hispanic Black respondents reported considerably fewer hours of sleep yet better sleep quality.

For ease of interpretation, Table 4 presents the predicted estimates of how sleep and health outcomes might vary by employment patterns based on the results reported in Tables 2 and 3. Across all outcomes, among those employed, individuals engaged in the “early ST-volatile” pattern between 22 and 49 had fewer (if not the fewest) hours of sleep (6.80 hrs/day), the lowest quality of sleep (-0.02), the highest likelihood of self-reporting poor health (0.23), the lowest SF-12 physical and mental functions scores (48.62 and 52.45), and the highest likelihood of having depressive symptoms (0.19). In contrast, individuals engaged in the “stable ST” pattern had the best outcomes, followed by those with the pattern of “mostly ST with some VH.”

**Table 4:** Predicted estimates of health outcomes by work schedule pattern between ages 22 and 48/49.

	Average sleep hours per day/wk	Sleep Quality	Poor Health	SF-12 Physical Function	SF-12 Mental Function	CES-D
Mostly NW	6.89 (0.07) [6.75, 7.02]	-0.01 (0.04) [-0.09, 0.06]	0.21 (0.01) [0.18, 0.24]	48.08 (0.48) [47.13, 49.02]	52.62 (0.42) [51.81, 53.44]	0.17 (0.01) [0.15, 0.20]
Early ST-Mostly VH	6.80 (0.04) [6.71, 6.89]	0.01 (0.03) [-0.04, 0.06]	0.18 (0.01) [0.15, 0.20]	49.15 (0.34) [48.48, 49.82]	53.07 (0.29) [52.50, 53.63]	0.17 (0.01) [0.14, 0.19]
Early ST-Volatile	6.80 (0.04) [6.71, 6.88]	-0.02 (0.02) [-0.06, 0.02]	0.23 (0.01) [0.21, 0.25]	48.62 (0.28) [48.06, 49.18]	52.45 (0.26) [51.94, 52.96]	0.19 (0.01) [0.17, 0.21]
Mostly ST with some VH	6.93 (0.03) [6.88, 6.99]	0.01 (0.02) [-0.02, 0.04]	0.19 (0.01) [0.18, 0.21]	49.43 (0.19) [49.06, 49.80]	53.02 (0.17) [52.68, 53.36]	0.17 (0.01) [0.15, 0.18]
Stable ST	7.03 (0.03) [6.97, 7.09]	0.06 (0.02) [0.02, 0.09]	0.17 (0.01) [0.15, 0.19]	50.04 (0.20) [49.64, 50.44]	53.27 (0.18) [52.91, 53.64]	0.14 (0.01) [0.12, 0.16]

*Note*. ST: standard hours; VH: variable hours; NW: not working; Numbers represent probabilities based on results reported in Tables 2 and 3 with delta-method standard errors in parentheses and 95% confidence interval in brackets.

### Variations in links between employment patterns and outcomes by social position

Prior research suggests that social position influences employment patterns, with vital implications for our health [3, 5, 6, 17, 18]. Figs 2–7 display the predicted estimates of the six outcomes by intersecting employment patterns, gender, race, and education. S2–S7 Tables present the predicted estimates in detail. Several findings are worth highlighting. First, education serves an important cushion for better sleep (hours and quality) and health outcomes regardless of employment pattern, race, or gender. Second, the significantly poorer sleep and health outcomes observed in Tables 2 and 3 were concentrated among people with vulnerable positions, such as females, racial minorities (with some exceptions detailed below), and those with less than a college degree. For example, Black males with the “early ST-mostly VH” employment pattern slept the least regardless of education; their average sleep hours were 6.39, 6.44, 6.44, and 6.50, respectively, for less than a high school degree, high school, some college, and college and above. On the other spectrum are White females, who tended to have the most sleep hours, particularly if they had the employment pattern of stable standard hours (7.14, 7.19, 7.19, and 7.25 for the educational groups of less than a high school degree to college or above) (Fig 2 and S2 Table).

In contrast with the sleep hours results, Black males reported the best and White females the worst sleep quality. These findings are particularly true for those with either the “early ST-volatile” or the “mostly ST with some VH” employment pattern regardless of educational attainment (Fig 3 and S3 Table).

The health outcomes also varied by employment pattern and social position. In general, “early ST-volatile” reported the poorest health outcomes across all educational groups and all racial/ethnic and gender pairings. Specifically, across all education categories, Black females who had the "early ST-volatile" employment pattern reported the highest likelihood of having poor health among all groups examined. Despite White males in the “early ST-volatile” group also reporting a high likelihood of having poor health, the difference in the likelihood of reporting poor health between Black females (.34) and White males (.27) with this employment pattern is about .07 among those with less than a high school education (Fig 4 and S4 Table). In addition, Black females with the “early ST-volatile” employment pattern had the lowest SF-12 physical function score, whereas males, regardless of race, reported the highest SF-12 physical function (Fig 5 and S5 Table). In regard to SF-12 mental function, Black males and females generally reported better scores than White males and females. In addition, White females with the “early ST-volatile” employment pattern reported the lowest SF-12 mental function; this is particularly true if they had a less than high school education (Fig 6 and S6 Table).

Similar to the SF-12 mental health results, Black males and females generally reported a lower likelihood of having depressive symptoms than their White counterparts. In addition, White females with the “early ST-volatile” employment pattern reported the highest likelihood of having depressive symptoms, particularly among those with less than a high school education (Fig 7 and S7 Table). The difference in the likelihood of having depressive symptoms between this group and White males in the highest education group with the “stable ST” employment patterns is striking: .32 versus .07.

## Discussion and conclusion

Since the 1980s, our employment has been shaped by global technological and digital advances, together with the rise and dominance of the service economy. These changes have produced undesirable health consequences, including disrupting our sleep routines, an aspect of our daily life critical to nurturing our health. Decades of research has established that sleep, both duration and quality, matters to our health [5]. This paper contributes two crucial insights to advance our knowledge of how work may have become a vulnerability for our sleep and health. Specifically, nonstandard work schedules, a central indicator of precarious employment, have become a widespread job characteristic in the increasingly unequal and globalized labor market [3, 15]. Moreover, the strains and harm caused by the recent devastating public health crisis (the COVID-19 pandemic) were disproportionately carried by those without resources and those with precarious jobs [51], particularly in the United States [6]. This study thus examines the extent to which having a nonstandard work schedule throughout one’s working life in the United States might make a difference in both sleep hours and quality and health outcomes. I paid particular attention to the relationship between employment patterns, sleep, and health outcomes among the groups most likely to be subject to working nonstandard hours. Below I highlight a few significant findings.

Using a nationally representative, longitudinal sample of U.S. individuals interviewed since 1979, this study finds that employment patterns over our working lives matter to our sleep and health, consistent with prior research [1, 5, 12, 52]. Importantly, this study approaches this issue from a life-course perspective, examining how employment patterns over our working lives might be linked to our sleep and health by shaping our daily routines. My empirical results suggest that individuals engaged in volatile work schedule patterns—a combination of evening, night, and variable hours—could anticipate sleeping significantly fewer hours per day, getting lower sleep quality, perceiving lower SF-12 physical and mental functions, and reporting a higher likelihood of poor health and depressive symptoms at age 50 than people working regular daytime hours. In fact, any employment pattern involving nonstandard hours (such as evening, night, or variable hours) for most of one’s working years may be associated with adverse sleep and health outcomes. These results suggest that a job requiring constant changes between daytime, evenings, nights, and irregular hours could significantly interfere with daily routines, affecting when a person sleeps, eats, and socializes with family members and friends. Furthermore, night shifts require a waking state during night hours when our bodies need rest, disrupting our circadian rhythm and thus sleep routines, including sleep quality. The lack of (good quality) sleep, physical fatigue, and emotional exhaustion stemming from a volatile employment pattern exemplifies how our work has made us vulnerable to an unhealthy life. Indeed, in regard to the SF-12 physical and mental function scores and the likelihood of having depressive symptoms, the effect sizes associated with the “early ST-volatile” employment pattern were similar to, if not larger than, having less than a high school education (see Table 3). This adverse health consequence of nonstandard work schedule patterns is alarming given that the extant research has shown that getting an inadequate amount of sleep and having poor sleep quality can have myriad short- and long-term health consequences, ranging from somatic issues and increased stress responsivity, which can lead to increased anxiety and depression [53], to a high prevalence of hypertension, obesity, and stroke [5].

The picture becomes grimmer if we further disentangle these links by social position. For example, as shown in Table 1, Blacks were more likely than their White peers to have an employment pattern of starting with standard hours but soon transitioning into volatile schedules for most of their working years. Importantly, the intersectionality between employment patterns and social position only underscores the substantial health disparities between those with resources and those without: those without disproportionately shoulder the adverse consequences of employment patterns characterized by volatility, confirming that advantages and disadvantages produced by our work can accumulate throughout a lifetime, with powerful implications for our health and well-being. The empirical evidence reported here shows that White females with a college or above education who had an employment pattern of stable standard-hour schedules (“stable-ST”) got on average six more hours of sleep a week ((7.25–6.39) x 7) than Black males with less than a high school degree who worked variable hours for most of their working years (“early ST-mostly VH”). Even within the group with less than a high school education, White females with the “stable-ST” employment pattern got on average five more hours of sleep a week ((7.14–6.39) x 7) than Black males with the “early ST-mostly VH” employment pattern. Similarly, the likelihood of reporting poor health was .09 among White males with a college or above education and an employment pattern of stable standard hours versus .34 (the highest likelihood) among Black females with a less than high school education and the “early ST-volatile” employment pattern. The former also reported significantly better SF-12 physical function than the latter, with an effect size of five-tenths of one standard deviation (51.61–46.44 = 5.17) (see S5 Table).

However, the opposite is true regarding gender differences in sleep quality and mental health. Specifically, females generally reported more hours of sleep but also poorer sleep quality than their male counterparts, which is consistent with the established scholarship in this area [54, 55]. Studies have examined whether gender differences in sleep quality might be related to biological (e.g., genetics) and sociological (e.g., family responsibilities, work) factors [54]. The extant research suggests that family responsibilities and work characteristics are the most important factors explaining why women experience sleep disorders more than men [54]. Specifically, among those who work nonstandard schedules, women are more likely to have sleep disorders than men [54]. In line with the literature, my analyses show that (see S3 Table) White and Black females with less than a high school degree who had volatile employment patterns between ages 22 and 49 reported a sleep quality of -.29 and -.12, respectively. In contrast, the corresponding estimates were -.10 and .05 for White and Black males with that same educational level and employment pattern. As the sleep quality variable is a standardized score with a mean of 0 and a standard deviation of 1, the differences between White women and Black men amount to a one-third of a standard deviation (e.g., -.29 –(.05) / 1 = .34) among those with less than a high school degree and the employment pattern of “early ST-volatile.” Further, when comparing White and Black males with a college degree and the “stable ST” employment pattern, the corresponding estimates were .18 and .25. The difference between White females with a less than a high school degree and an employment pattern of “early ST-volatile” and Black males with a college or above education and a “stable ST” employment pattern is even larger, amounting to slightly over half of a standard deviation (e.g., -.29 –(.25) / 1 = .54). These differences are considered medium to large effect sizes [55, 56].

In regard to mental health, extant studies have shown that males are less likely to report mental health symptoms than females, a finding echoed in my analysis. Prior research indicates that although males and females were equally likely to experience emotional stress, males were less likely than females to express stress in ways that are measured through items in the SF-12 or CES-D instruments [57]. In addition, studies have found that females are more likely than males to report mild-moderate depression, but males report severe depression and suicidal thoughts more often than females [58]. Because the measures used in this study assess mild to moderate depressive symptoms, my findings that females reported poorer mental functions and a higher likelihood of having at-risk depressive symptoms than males are in line with previous empirical evidence. Furthermore, prior research has indicated that gender differences in symptom phenotypes (e.g., atypical symptoms in male depression) or in coping style (e.g., males tend not to seek help) are mechanisms that might explain why studies tend to observe a higher incidence of depression among females than males [58].

In contrast, and importantly, the racial differences in sleep hours and quality, and in health outcomes found in my analyses are nuanced and far from straightforward. Although Black males and, to a lesser extent, Black females tended to report similar physical and mental health as their White counterparts, the contrasts are striking when looking at sleep-related results. Specifically, Black males reported the fewest hours of sleep yet the best sleep quality, whereas White females tended to report more sleep hours but much poorer sleep quality. Although the extant research suggests that Blacks tend to sleep fewer hours (e.g., < 7 hrs) and have poorer sleep quality than their White counterparts, the Black–White disparities in sleep quality previously documented are somewhat mixed [59]. For example, studies that use objective measurements to assess sleep quality tend to confirm the Black–White disparities in sleep quality [59]. However, the results are less definitive when a subjective measure such as self-reports are used [59], which is how sleep quality was collected in the NLSY79. Prior studies have also shown that the Black–White disparities in sleep quality might have to do with socioeconomic status or environmental factors (e.g., neighborhood quality) [59–61]. In other words, the Black–White disparities in sleep quality might disappear once we consider these factors. Indeed, the raw data of this analysis indicated that Blacks in the NLSY79 sample reported the lowest sleep quality among all respondents, but this disparity disappeared in the multiple regression analysis when a rich set of sociodemographic characteristics were considered, including their work schedule trajectories.

Although beyond the scope of this paper, the scholarship on the “black–white health paradox” might also corroborate my findings that the Black respondents tended to report better sleep quality and similar if not better physical and mental functions despite shorter sleep duration [62]. Social stress theory, and related approaches, would predict that racial minority groups in the United States like Black Americans should be more likely than their White peers to develop poor physical and mental health due to discrimination-related experiences, in line with the core assumption of CAD [63]. However, prior studies using self-reported data (the same method as used with the NLSY79) have documented that Blacks display similar physical health and better mental health than their White counterparts [64, 65]. Researchers have posited that experiencing discrimination, hardship, and stresses may increase resilience in the face of challenges [63, 65]. If so, nonstandard work schedules, considered a disadvantage, might not directly translate into poor health for Blacks. However, caution is warranted when making any sweeping generalizations based on my results. After all, the predicted estimates shown in S3 Table suggest that Black males and females with less than a high school degree and a volatile employment pattern for most of their working lives had a high, if not the highest, likelihood of reporting poor health (.29 and .34, respectively) among all respondents. This association between perceived poor health and the joint forces of work and social position warrants attention in future research and policy advocacy endeavors.

### Limitations

As with all observational studies, the current study has several limitations. First, the NLSY79 provided work schedule information annually until 1994 and biennially thereafter. For some, work schedules may have changed from month to month, let alone during the two-year windows, limiting my ability to depict more precise employment patterns over time. Thus, the present results may underestimate the true association between employment patterns and outcomes. However, the longitudinal approach has the advantage of reducing measurement noise. Specifically, longitudinal data allow more accuracy than cross-sectional data in recognizing, for example, individuals who have repeatedly reported nonstandard work schedules over the years versus those who might have only worked such a schedule a few times over 30 years.

Second, our daily routines and health are closely related to the type and amount of resources we can access; income and wages are the primary means of securing such resources. Ideally, the true association between employment patterns and sleep and health would be obtained after considering income and wages. However, the high correlation between employment and income and wages creates concerns about reverse causality. After all, our type of work determines how much income we can bring home. To address this potential reverse-causality issue, I controlled for the following variables likely to shape the type of work one may access at the start of their career and thus the wages and income they might bring home: the educational levels of the respondents and their parents and whether they had ever experienced poverty and/or received welfare before age 22. Nonetheless, our knowledge will benefit from a closer examination of how wages and income might play a critical role in the association between employment patterns and our sleep and health. For example, although it is beyond the scope of this analysis, future research might pursue this research question by utilizing a structural equation model to establish a proper temporal order between employment patterns (e.g., between ages 22 and 40), wages and income (e.g., between ages 41 and 49), and health outcomes (e.g., at age 50) to avoid reverse-causality issues. In this study, I did not adopt a structural equation modeling as specified above because my primary aim was to build upon a life-course lens to document the respondents’ work schedule trajectories using as many working years as possible (i.e., between ages 22 and 49 vs. between ages 22 and 39). Similarly, due to the data at hand, this analysis could not consider the number of hours respondents spent on household chores, which plays an important role not only in how many hours we can sleep but also in our physical and mental health. For example, women generally spend more time doing household work than men do despite potentially having the same number of working hours. These differences in household chores influence the number of hours a woman versus a man has for sleep and can impact their physical and emotional energy levels.

Third, individuals may switch from evening/night hours to standard daytime hours due to worsening health stemming from working nonstandard hours. If so, the estimates of the links between employment patterns and the sleep and health outcomes might be underestimated in this analysis. The sequence analysis adopted in this paper does not sufficiently answer such research questions. Although it is beyond the scope of this paper, future research might use other appropriate statistical analyses (e.g., latent transition analysis) to examine this crucial dynamic nature between employment patterns and sleep and health over time. Similarly, fixed-effect models might help answer research questions about how changes in employment patterns may shape changes in sleep and health over time. Such analyses would require at least three data points of health outcomes. This analysis relies on the 2018 NLSY79 data release, at which point only two such data points were available for the majority of its sample, as only about 10% of the respondents had reached age 60 to have three data points of health outcomes. In addition, the NLSY79 collected the sleep information analyzed here only in the health module at ages 50 and 60, thus limiting the ability to conduct the more sophisticated statistical analyses needed to answer more dynamic research questions.

Fourth, despite the sequence analysis accurately documenting the sequential changes in work trajectories, the analyses presented here at best represent associations instead of causation. While an experimental design study might allow one to detect a causal relationship between employment and sleep and health, randomly assigning individuals to different employment and various work schedule patterns would be neither feasible nor ethical. Hence, relying on quality longitudinal data with proper and sophisticated statistical analysis (e.g., fixed-effect modeling, instrumental variable) would allow us one step closer to causation.

Fifth, most of the NLSY79 health variables are self-reported, which likely influenced the outcomes identified here. Knowing how individuals perceive their sleep quality and health outcomes is critical as subjective perceptions significantly affect our well-being. However, the extant research using objective measurement tools consistently confirms a Black–White disparity in sleep quality and health outcomes, highlighting the importance of triangulating information to increase confidence in the findings.

Sixth, sample attrition is unavoidable with longitudinal data and could have affected some results. The positive selection bias associated with sample attrition might also bias the true association between employment patterns and sleep and health outcomes.

Seventh, although I used a separate racial-ethnic group named “other” that included Asian and other ethnicities, the estimates suffer from extremely small sample sizes, prohibiting me from drawing definitive interpretations about this group. This limitation warrants attention in future efforts to collect nationally representative data. Despite these limitations, the NLSY79 is the only dataset containing work schedule information for a nationally representative sample in the U.S. over three decades during a period when nonstandard work schedules were increasingly becoming prevalent throughout the country.

### Conclusion

This study uses a life-course lens to shed much-needed light on how our employment patterns might shape our sleep and health as we approach middle adulthood. Employment is a crucial factor in the process of producing and accumulating resources and risks throughout our lives. Of importance, precarious employment has become increasingly typical in the globalized and polarized labor market, and nonstandard work schedules are a critical feature of precarious jobs. Examining employment patterns and work schedules through a longitudinal lens thus provides a deeper appreciation of how the impact of nonstandard work schedules might manifest through advantages and disadvantages accumulated throughout one’s working life. This approach also underscores that the health burden might be disproportionately shouldered by workers in vulnerable social positions (e.g., females, low education, Blacks). This study’s findings highlight the dual challenges facing workers in vulnerable social positions who have jobs requiring nonstandard work schedules, both of which limit their access to resources that would allow them to achieve decent sleep health and physical and mental health outcomes. This analysis thus calls attention to the reality of how employment as a social system may generate and perpetuate vulnerabilities and inequalities for particular groups over the life course.

## Supporting information

S1 Table Goodness-of-fit statistics for work arrangements sequence cluster solutions. (DOCX)

S2 Table Adjusted predictions of average sleep hours per day/week at age 50 by work schedule patterns, gender, race, and education. (DOCX)

S3 Table Adjusted predictions of sleep quality at age 50 by work schedule patterns, gender, race, and education. (DOCX)

S4 Table Adjusted predicted probabilities of self-reporting poor health at age 50 by work schedule patterns, gender, race, and education. (DOCX)

S5 Table Adjusted predictions of SF-12 physical function at age 50 by work schedule patterns, gender, race, and education. (DOCX)

S6 Table Adjusted predictions of SF-12 mental function at age 50 by work schedule patterns, gender, race, and education. (DOCX)

S7 Table Adjusted predicted probabilities of self-reporting depressive symptoms at age 50 by work schedule patterns, gender, race, and education. (DOCX)
