# Supplementary material for: How our longitudinal employment patterns might shape our health as we approach middle adulthood—US NLSY79 cohort
Source: PLoS One. 2024 Apr 3;19(4):e0300245. doi: 10.1371/journal.pone.0300245 (PMC10990189; doi:10.1371/journal.pone.0300245)
Supplement: S1 Table — (DOCX) [file pone.0300245.s001.docx]

**S1 Table. Goodness-of-Fit Statistics for Work Arrangements Sequence Cluster Solutions**

|  | Calinski/Harabasz pseudo-F | Duda/Hart | |
| --- | --- | --- | --- |
| Number of Clusters |  | Je(2)/Je(1) | Pseudo T-squared |
| 2 | 11.97 | 0.9984 | 9.14 |
| 3 | 10.53 | 0.9968 | 7.74 |
| 4 | 9.62 | 0.9983 | 4.92 |
| 5 | 8.43 | 0.9997 | 0.32 |
| 6 | 6.81 | 0.9997 | 0.74 |
| 7 | 5.79 | 0.9962 | 7.02 |
| 8 | 5.98 | 0.9896 | 8.09 |
| 9 | 6.25 | 0.9937 | 4.55 |
| 10 | 6.03 | 0.9998 | 0.16 |
| 11 | 5.44 | 0.9978 | 2.72 |
| 12 | 5.20 | 0.9963 | 7.08 |
| 13 | 5.38 | 0.9998 | 0.12 |
| 14 | 4.97 | 0.9967 | 3.45 |
| 15 | 4.87 | 0.9951 | 4.18 |

*Note*. The two stopping-rule values for each cluster solution are presented above, representing the Calinski and Harabasz pseudo-F index and the Duda-Hart Je(2)/Je(1) index. In both indices, larger values indicate a more distinct cluster, whereas smaller pseudo-T-squared values indicate more distinct clustering (Milligan & Cooper, 1985).

Milligan GW and Cooper MC. An examination of procedures for determining the number of clusters in a data set. Psychometrika. 1985; 50(2): 159–179.
