# Supplementary material for: How our longitudinal employment patterns might shape our health as we approach middle adulthood—US NLSY79 cohort
Source: PLoS One. 2024 Apr 3;19(4):e0300245. doi: 10.1371/journal.pone.0300245 (PMC10990189; doi:10.1371/journal.pone.0300245)
Supplement: S2 Table — (DOCX) [file pone.0300245.s002.docx]

**S2 Table. Adjusted Predictions of Average Sleep Hours Per Day/Week at Age 50 by Work Schedule Patterns, Gender, Race, and Education**

|  | Mostly NW | Early ST-Mostly VH | Early ST-Volatile | Mostly ST with some VH | Stable ST |
| --- | --- | --- | --- | --- | --- |
| *Less than High School* |  |  |  |  |  |
| Non-Hispanic White Male | 6.78 [6.30, 7.26] | 6.78 [6.60, 6.95] | 6.71 [6.54, 6.89] | 6.81 [6.67, 6.95] | 6.96 [6.84, 7.09] |
| Non-Hispanic Black Male | 6.59 [6.18, 6.99] | 6.39 [6.12, 6.65] | 6.63 [6.42, 6.83] | 6.85 [6.66, 7.04] | 6.88 [6.67, 7.08] |
| Non-Hispanic White Female | 6.90 [6.68, 7.12] | 6.97 [6.79, 7.15] | 6.91 [6.67, 7.13] | 6.92 [6.78, 7.05] | 7.14 [7.00, 7.29] |
| Non-Hispanic Black Female | 6.91 [6.61, 7.21] | 6.61 [6.28, 6.94] | 6.64 [6.39, 6.88] | 6.86 [6.66, 7.07] | 6.70 [6.49, 6.90] |
| *High School* |  |  |  |  |  |
| Non-Hispanic White Male | 6.83 [6.35, 7.30] | 6.82 [6.67, 6.97] | 6.76 [6.60, 6.92] | 6.86 [6.74, 6.98] | 7.01 [6.91, 7.11] |
| Non-Hispanic Black Male | 6.63 [6.24, 7.02] | 6.44 [6.18, 6.68] | 6.67 [6.49, 6.86] | 6.90 [6.72, 7.07] | 6.92 [6.73, 7.11] |
| Non-Hispanic White Female | 6.95 [6.74, 7.16] | 7.02 [6.85, 7.18] | 6.96 [6.75, 7.15] | 6.96 [6.85, 7.07] | 7.19 [7.07, 7.30] |
| Non-Hispanic Black Female | 6.96 [6.68, 7.24] | 6.66 [6.34, 6.98] | 6.68 [6.46, 6.90] | 6.91 [6.73, 7.09] | 6.74 [6.56, 6.92] |
| *Some College* |  |  |  |  |  |
| Non-Hispanic White Male | 6.83 [6.35, 7.31] | 6.83 [6.67, 6.98] | 6.76 [6.60, 6.93] | 6.86 [6.74, 6.99] | 7.02 [6.90, 7.13] |
| Non-Hispanic Black Male | 6.64 [6.24, 7.03] | 6.44 [6.19, 6.69] | 6.68 [6.49, 6.86] | 6.90 [6.72, 7.08] | 6.93 [6.73, 7.12] |
| Non-Hispanic White Female | 6.95 [6.74, 7.17] | 7.02 [6.85, 7.19] | 6.96 [6.75, 7.17] | 6.97 [6.85, 7.09] | 7.19 [7.07, 7.32] |
| Non-Hispanic Black Female | 6.96 [6.68, 7.24] | 6.66 [6.34, 6.98] | 6.69 [6.47, 6.91] | 6.92 [6.74, 7.09] | 6.75 [6.57, 6.93] |
| *College+* |  |  |  |  |  |
| Non-Hispanic White Male | 6.89 [6.42, 7.37] | 6.89 [6.72, 7.05] | 6.83 [6.65, 7.01] | 6.93 [6.79, 7.06] | 7.08 [6.95, 7.20] |
| Non-Hispanic Black Male | 6.70 [6.30, 7.10] | 6.50 [6.24, 6.76] | 6.74 [6.55, 6.94] | 6.96 [6.77, 7.15] | 6.99 [6.78, 7.20] |
| Non-Hispanic White Female | 7.02 [6.80, 7.23] | 7.08 [6.91, 7.26] | 7.02 [6.81, 7.24] | 7.03 [6.90, 7.16] | 7.25 [7.13, 7.38] |
| Non-Hispanic Black Female | 7.02 [6.74, 7.31] | 6.73 [6.40, 7.05] | 6.75 [6.52, 6.98] | 6.98 [6.79, 7.16] | 6.81 [6.62 7.00] |

*Note*. ST: standard hours; VH: variable hours; NW: not working. Numbers represented predicted average sleep hours per day during a 7-day week based on regression results reported in Table 2-1 with 95% confidence intervals shown in brackets.
