# Supplementary material for: How our longitudinal employment patterns might shape our health as we approach middle adulthood—US NLSY79 cohort
Source: PLoS One. 2024 Apr 3;19(4):e0300245. doi: 10.1371/journal.pone.0300245 (PMC10990189; doi:10.1371/journal.pone.0300245)
Supplement: S3 Table — (DOCX) [file pone.0300245.s003.docx]

**S3 Table. Adjusted Predictions of Sleep Quality at Age 50 by Work Schedule Patterns, Gender, Race, and Education**

|  | Mostly NW | Early ST-Mostly VH | Early ST-Volatile | Mostly ST with some VH | Stable ST |
| --- | --- | --- | --- | --- | --- |
| *Less than High School* |  |  |  |  |  |
| Non-Hispanic White Male | .03 [-.20, .26] | -.06 [-.17, .04] | -.10 [-.20, .01] | -.08 [-.17, -.00] | -.02 [-.09, .06] |
| Non-Hispanic Black Male | -.01 [-.19, .16] | -.07 [-.23, .09] | .05 [-.06, .16] | .05 [-.04, .15] | .05 [-.06, .16] |
| Non-Hispanic White Female | -.26 [-.38, -.14] | -.18 [-.29, -.06] | -.29 [-.41, -.18] | -.23 [-.31, -.15] | -.17 [-.26, -.09] |
| Non-Hispanic Black Female | -.08 [-.23, .08] | -.03 [-.22, .16] | -.12 [-.25, .00] | -.09 [-.19, .02] | -.07 [-.20, .05] |
| *High School* |  |  |  |  |  |
| Non-Hispanic White Male | .14 [-.08, .37] | .05 [-.04, .15] | .02 [-.08, .11] | .03 [-.04, .10] | .10 [.03, .16] |
| Non-Hispanic Black Male | .10 [-.07, .27] | .04 [-.11, .20] | .16 [.07, .26] | .17 [.08, .25] | .17 [.06, .28] |
| Non-Hispanic White Female | -.14 [-.26, -.03] | -.06 [-.17, .04] | -.18 [-.28, -.07] | -.11 [-.18, -.05] | -.06 [-.13, .01] |
| Non-Hispanic Black Female | .04 [-.11, .18] | .08 [-.10, .27] | -.01 [-.12, .10] | .03 [-.06, .12] | .04 [-.08, .15] |
| *Some College* |  |  |  |  |  |
| Non-Hispanic White Male | .15 [-.08, .38] | .06 [-.04, .15] | .10 [-.01, .21] | .03 [-.04, .11] | .10 [.03, .17] |
| Non-Hispanic Black Male | .10 [-.07, .28] | .05 [-.11, .21] | .17 [.07, .27] | .17 [.08, .26] | .17 [.07, .28] |
| Non-Hispanic White Female | -.14 [-.25, -.03] | -.06 [-.17, .05] | -.17 [-.28, -.06] | -.11 [-.18, -.04] | -.06 [-.13, .02] |
| Non-Hispanic Black Female | .04 [-.11, .19] | .09 [-.10, .27] | -.00 [-.12, .11] | .03 [-.06, .12] | .04 [-.07, .16] |
| *College+* |  |  |  |  |  |
| Non-Hispanic White Male | .23 [-.00, .46] | .13 [.03, .24] | .05 [-.06, .16] | .11 [.03, .19] | .18 [.10, .26] |
| Non-Hispanic Black Male | .18 [.00, .36] | .13 [-.04, .29] | .25 [.14, .35] | .25 [.15, .35] | .25 [.13, .36] |
| Non-Hispanic White Female | -.06 [-.18, .06] | .02 [-.10, .13] | -.10 [-.21, .02] | -.03 [-.11, .05] | .02 [-.05, .10] |
| Non-Hispanic Black Female | .12 [-.03, .27] | .17 [-.02, .35] | .07 [-.04, .19] | .11 [.01, .21] | .12 [.00, .24] |

*Note*. ST: standard hours; VH: variable hours; NW: not working. Numbers represented predicted sleep quality based on regression results reported in Table 2-1 with 95% confidence intervals shown in brackets.
