# Supplementary material for: How our longitudinal employment patterns might shape our health as we approach middle adulthood—US NLSY79 cohort
Source: PLoS One. 2024 Apr 3;19(4):e0300245. doi: 10.1371/journal.pone.0300245 (PMC10990189; doi:10.1371/journal.pone.0300245)
Supplement: S4 Table — (DOCX) [file pone.0300245.s004.docx]

**S4 Table. Adjusted Predicted Probabilities of Self-Reporting Poor Health at Age 50 by Work Schedule Patterns, Gender, Race, and Education**

|  | Mostly NW | Early ST-Mostly VH | Early ST-Volatile | Mostly ST with some VH | Stable ST |
| --- | --- | --- | --- | --- | --- |
| *Less than High School* |  |  |  |  |  |
| Non-Hispanic White Male | .26 [.14, .37] | .22 [.12, .29] | .27 [.21, .34] | .24 [.19, .28] | .22 [.18, .27] |
| Non-Hispanic Black Male | .27 [.18, .35] | .26 [.17, .35] | .29 [.22, .35] | .27 [.21, .33] | .24 [.18, .31] |
| Non-Hispanic White Female | .30 [.23, .37] | .25 [.19, .32] | .29 [.22, .35] | .23 [.19, .28] | .20 [.16, .25] |
| Non-Hispanic Black Female | .23 [.17, .30] | .24 [.16, .34] | .34 [.27, .42] | .24 [.19, .29] | .23 [.16, .30] |
| *High School* |  |  |  |  |  |
| Non-Hispanic White Male | .20 [.11, .29] | .17 [.12, .22] | .22 [.16, .26] | .18 [.15, .22] | .17 [.14, .21] |
| Non-Hispanic Black Male | .21 [.14, .28] | .21 [.13, .28] | .23 [.18, .28] | .21 [.17, .25] | .19 [.14, .25] |
| Non-Hispanic White Female | .24 [.18, .29] | .20 [.14, .25] | .23 [.17, .28] | .18 [.15, .21] | .16 [.12, .20] |
| Non-Hispanic Black Female | .18 [.13, ,24] | .19 [.12, .26] | .28 [.21, .34] | .19 [.15, .23] | .18 [.12, .24] |
| *Some College* |  |  |  |  |  |
| Non-Hispanic White Male | .18 [.09, .26] | .15 [.10, .20] | .19 [.14, .24] | .16 [.12, .20] | .15 [.12, .19] |
| Non-Hispanic Black Male | .18 [.12, .25] | .18 [.11, .25] | .20 [.15, .25] | .19 [.14, .23] | .17 [.12, .22] |
| Non-Hispanic White Female | .21 [.15, .26] | .17 [.12, .22] | .20 [.15, .25] | .16 [.13, .19] | .14 [.10, .17] |
| Non-Hispanic Black Female | .16 [.11, .21] | .17 [.10, .23] | .25 [.18, .31] | .16 [.13, .20] | .15 [.10, .21] |
| *College+* |  |  |  |  |  |
| Non-Hispanic White Male | .10 [.04, .16] | .09 [.05, .12] | .11 [.07, .15] | .09 [.06, .12] | .09 [.06, .12] |
| Non-Hispanic Black Male | .11 [.06, .16] | .11 [.06, .16] | .12 [.08, .16] | .11 [.07, .15] | .10 [.06, .14] |
| Non-Hispanic White Female | .13 [.08, 178] | .10 [.06, .14] | .12 [.08, .16] | .09 [.06, .12] | .08 [.05, .11] |
| Non-Hispanic Black Female | .09 [.06, .13] | .10 [.05, .15] | .15 [.10, .21] | .10 [.06, .13] | .09 [.05, .13] |

*Note*. ST: standard hours; VH: variable hours; NW: not working. Numbers represented predicted probabilities of reporting poor health based on regression results reported in Table 2-1 with 95% confidence intervals shown in brackets.
