# Supplementary material for: How our longitudinal employment patterns might shape our health as we approach middle adulthood—US NLSY79 cohort
Source: PLoS One. 2024 Apr 3;19(4):e0300245. doi: 10.1371/journal.pone.0300245 (PMC10990189; doi:10.1371/journal.pone.0300245)
Supplement: S5 Table — (DOCX) [file pone.0300245.s005.docx]

**S5 Table. Adjusted Predictions of SF-12 Physical Function at Age 50 by Work Schedule Patterns, Gender, Race, and Education**

|  | Mostly NW | Early ST-Mostly VH | Early ST-Volatile | Mostly ST with some VH | Stable ST |
| --- | --- | --- | --- | --- | --- |
| *Less than High School* |  |  |  |  |  |
| Non-Hispanic White Male | 47.04 [44.47, 49.60] | 48.54 [47.32, 49.76] | 47.63 [46.40, 48.86] | 48.13 [47.14, 49.12] | 48.81 [47.93, 49.69] |
| Non-Hispanic Black Male | 48.49 [46.33, 50.66] | 48.11 [46.18, 50.04] | 48.47 [47.08, 49.86] | 48.95 [47.76, 50.14] | 48.79 [47.41, 50.17] |
| Non-Hispanic White Female | 46.09 [44.43, 47.75] | 46.99 [45.49, 48.49] | 47.33 [45.89, 48.77] | 47.38 [46.36, 48.40] | 48.43 [47.42, 49.44] |
| Non-Hispanic Black Female | 47.17 [45.13, 49.21] | 47.97 [45.59, 50.34] | 46.44 [44.74, 48.14] | 49.28 [47.99, 50.58] | 48.21 [46.78, 49.63] |
| *High School* |  |  |  |  |  |
| Non-Hispanic White Male | 48.16 [45.68, 50.64] | 49.66 [48.57, 50.74] | 48.75 [47.64, 49.86] | 49.25 [48.42, 50.08] | 49.93 [49.25, 50.61] |
| Non-Hispanic Black Male | 49.62 [47.55, 51.68] | 49.23 [47.40, 51.06] | 49.59 [48.34, 50.84] | 50.07 [49.01, 51.13] | 49.91 [48.64, 51.18] |
| Non-Hispanic White Female | 47.21 [45.65, 48.77] | 48.11 [46.72, 49.50] | 48.45 [47.13, 49.78] | 48.50 [47.68, 49.32] | 49.55 [48.71, 50.39] |
| Non-Hispanic Black Female | 48.29 [46.35, 50.23] | 49.09 [46.80, 51.38] | 47.56 [46.00, 49.12] | 50.40 [49.33, 51.48] | 49.33 [48.05, 50.60] |
| *Some College* |  |  |  |  |  |
| Non-Hispanic White Male | 48.34 [45.83, 50.85] | 49.84 [48.72, 50.96] | 48.93 [47.78, 50.08] | 49.43 [48.55, 50.31] | 50.11 [49.33, 50.89] |
| Non-Hispanic Black Male | 49.80 [47.70, 51.90] | 49.41 [47.60, 51.23] | 49.77 [48.52, 51.03] | 50.25 [49.14, 51.37] | 50.09 [48.75, 51.43] |
| Non-Hispanic White Female | 47.40 [45.80, 49.00] | 48.30 [46.87, 49.72] | 48.64 [47.25, 50.02] | 48.68 [47.79, 49.57] | 49.73 [48.84, 50.62] |
| Non-Hispanic Black Female | 48.48 [46.54, 50.42] | 49.27 [46.97, 51.57] | 47.74 [46.18, 49.31] | 50.59 [49.51, 51.66] | 49.51 [48.25, 50.77] |
| *College+* |  |  |  |  |  |
| Non-Hispanic White Male | 49.84 [47.30, 52.38] | 51.34 [50.16, 52.51] | 50.43 [49.22, 51.64] | 50.93 [50.00, 51.86] | 51.61 [50.79, 52.43] |
| Non-Hispanic Black Male | 51.30 [49.15, 53.44] | 50.91 [49.05, 52.77] | 51.27 [49.96, 52.59] | 51.75 [50.57, 52.93] | 51.59 [50.22, 52.96] |
| Non-Hispanic White Female | 48.89 [47.29, 50.49] | 49.79 [48.30, 51.28] | 50.13 [48.72, 51.55] | 50.81 [49.25, 51.12] | 51.23 [50.31, 52.15] |
| Non-Hispanic Black Female | 49.97 [47.99, 51.96] | 50.77 [48.43, 53.11] | 49.24 [47.63, 50.86] | 52.09 [50.95, 53.22] | 51.01 [49.70, 52.32] |

*Note*. ST: standard hours; VH: variable hours; NW: not working. Numbers represented predicted scores of SF-12 physical function based on regression results reported in Table 2-2 with 95% confidence intervals shown in brackets.
