# Supplementary material for: How our longitudinal employment patterns might shape our health as we approach middle adulthood—US NLSY79 cohort
Source: PLoS One. 2024 Apr 3;19(4):e0300245. doi: 10.1371/journal.pone.0300245 (PMC10990189; doi:10.1371/journal.pone.0300245)
Supplement: S6 Table — (DOCX) [file pone.0300245.s006.docx]

**S6 Table. Adjusted Predictions of SF-12 Mental Function at Age 50 by Work Schedule Patterns, Gender, Race, and Education**

|  | Mostly NW | Early ST-Mostly VH | Early ST-Volatile | Mostly ST with some VH | Stable ST |
| --- | --- | --- | --- | --- | --- |
| *Less than High School* |  |  |  |  |  |
| Non-Hispanic White Male | 55.52 [53.36, 57.68] | 52.58 [51.48, 53.68] | 51.93 [50.76, 53.10] | 52.59 [51.69, 53.49] | 53.14 [52.34, 53.95] |
| Non-Hispanic Black Male | 51.99 [49.88, 54.10] | 53.54 [52.03, 55.05] | 53.54 [52.42, 54.67] | 53.76 [52.70, 54.81] | 53.14 [51.99, 54.29] |
| Non-Hispanic White Female | 50.87 [49.43, 52.31] | 51.59 [50.23, 52.96] | 49.86 [48.41, 51.31] | 50.94 [50.01, 51.86] | 50.99 [50.03, 51.96] |
| Non-Hispanic Black Female | 52.43 [50.65, 54.22] | 52.79 [50.85, 54.73] | 53.15 [51.83, 54.47] | 52.91 [51.77, 54.06] | 53.05 [51.90, 54.19] |
| *High School* |  |  |  |  |  |
| Non-Hispanic White Male | 56.24 [54.14, 58.33] | 53.30 [52.34, 54.26] | 52.64 [51.60, 53.69] | 53.31 [52.58, 54.04] | 53.86 [53.22, 54.50] |
| Non-Hispanic Black Male | 52.71 [50.68, 54.74] | 54.26 [52.84, 55.68] | 54.26 [53.28, 55.24] | 54.48 [53.52, 55.43] | 53.86 [52.80, 54.92] |
| Non-Hispanic White Female | 51.59 [50.25, 52.93] | 52.31 [51.08, 53.54] | 50.57 [49.21, 51.94] | 51.65 [50.87, 52.43] | 51.71 [50.90, 52.52] |
| Non-Hispanic Black Female | 53.15 [51.48, 54.82] | 53.50 [51.63, 55.38] | 53.87 [52.70, 55.04] | 53.63 [52.66, 54.59] | 53.76 [52.78, 54.75] |
| *Some College* |  |  |  |  |  |
| Non-Hispanic White Male | 56.22 [54.10, 58.35] | 53.28 [52.29, 54.28] | 52.63 [51.56, 53.70] | 53.30 [52.51, 54.08] | 53.84 [53.12, 54.57] |
| Non-Hispanic Black Male | 52.69 [50.62, 54.76] | 54.24 [52.81, 55.68] | 54.25 [53.21, 55.28] | 54.46 [53.47, 55.46] | 53.84 [52.75, 54.93] |
| Non-Hispanic White Female | 51.57 [50.21, 52.94] | 52.29 [51.02, 53.56] | 50.56 [49.17, 51.95] | 51.64 [50.79, 52.48] | 51.70 [50.84, 52.56] |
| Non-Hispanic Black Female | 53.13 [51.45, 54.82] | 53.49 [51.60, 55.38] | 53.85 [52.67, 55.04] | 53.61 [52.66, 54.57] | 53.75 [52.74, 54.75] |
| *College+* |  |  |  |  |  |
| Non-Hispanic White Male | 56.33 [54.19, 58.48] | 53.40 [52.32, 54.47] | 52.74 [51.59, 53.88] | 53.41 [52.56, 54.25] | 53.96 [53.16, 54.75] |
| Non-Hispanic Black Male | 52.80 [50.69, 54.92] | 54.35 [52.84, 55.88] | 54.36 [53.26, 55.46] | 54.57 [53.50, 55.64] | 53.95 [52.81, 55.10] |
| Non-Hispanic White Female | 51.68 [50.29, 53.08] | 52.40 [51.08, 53.73] | 50.67 [49.24, 52.10] | 51.75 [50.86, 52.63] | 51.81 [50.92, 52.70] |
| Non-Hispanic Black Female | 53.24 [51.50, 54.98] | 53.60 [51.68, 55.52] | 53.97 [52.71, 55.22] | 53.72 [52.69, 54.76] | 53.86 [52.81, 54.91] |

*Note*. ST: standard hours; VH: variable hours; NW: not working. Numbers represented predicted scores of SF-12 mental function based on regression results reported in Table 2-2 with 95% confidence intervals shown in brackets.
