# Supplementary material for: How our longitudinal employment patterns might shape our health as we approach middle adulthood—US NLSY79 cohort
Source: PLoS One. 2024 Apr 3;19(4):e0300245. doi: 10.1371/journal.pone.0300245 (PMC10990189; doi:10.1371/journal.pone.0300245)
Supplement: S7 Table — (DOCX) [file pone.0300245.s007.docx]

**S7 Table. Adjusted Predicted Probabilities of Self-Reporting Depressive Symptoms at Age 50 by Work Schedule Patterns, Gender, Race, and Education**

|  | Mostly NW | Early ST-Mostly VH | Early ST-Volatile | Mostly ST with some VH | Stable ST |
| --- | --- | --- | --- | --- | --- |
| *Less than High School* |  |  |  |  |  |
| Non-Hispanic White Male | .18 [.08, .27] | .22 [.15, .28] | .24 [.18, .30] | .22 [.17, .26] | .14 [.10, .18] |
| Non-Hispanic Black Male | .21 [.13, .28] | .13 [.06, .19] | .13 [.08, .18] | .22 [.15, .29] | .16 [.11, .22] |
| Non-Hispanic White Female | .27 [.20, .34] | .25 [.18, .31] | .32 [.25, .39] | .28 [.23, .32] | .24 [.18, .29] |
| Non-Hispanic Black Female | .19 [.13, .25] | .22 [.13, .30] | .22 [.15, .29] | .19 [.14, .23] | .20 [.14, .27] |
| *High School* |  |  |  |  |  |
| Non-Hispanic White Male | .14 [.06, .22] | .18 [.13, .23] | .20 [.14, .25] | .18 [.14, .22] | .11 [.08, .14] |
| Non-Hispanic Black Male | .17 [.10, .23] | .10 [.05, .16] | .11 [.07, .15] | .11 [.07, .14] | .13 [.08, .18] |
| Non-Hispanic White Female | .23 [.17, .28] | .20 [.15, .26] | .27 [.21, .33] | .23 [.19, .26] | .19 [.15, .23] |
| Non-Hispanic Black Female | .15 [.10, .20] | .18 [.10, .25] | .18 [.13, .23] | .15 [.12, .19] | .17 [.11, .22] |
| *Some College* |  |  |  |  |  |
| Non-Hispanic White Male | .13 [.05, .20] | .16 [.11, .21] | .18 [.13, .22] | .16 [.12, .20] | .10 [.07, .13] |
| Non-Hispanic Black Male | .15 [.09, .21] | .09 [.04, .14] | .09 [.06, .13] | .10 [.06, .13] | .12 [.07, .16] |
| Non-Hispanic White Female | .20 [.15, .26] | .18 [.13, .24] | .25 [.19, .31] | .21 [.17, .24] | .17 [.13, .22] |
| Non-Hispanic Black Female | .14 [.09, .18] | .16 [.09, .23] | .16 [.11, .21] | .14 [.10, .17] | .15 [.10, .20] |
| *College+* |  |  |  |  |  |
| Non-Hispanic White Male | .09 [.03, .14] | .11 [.07, .15] | .12 [.08, .17] | .11 [.08, .15] | .07 [.04, .10] |
| Non-Hispanic Black Male | .10 [.05, .15] | .06 [.02, .10] | .06 [.04, .09] | .06 [.04, .09] | .08 [.04, .12] |
| Non-Hispanic White Female | .15 [.10, .19] | .13 [.08, .18] | .18 [.12, .23] | .15 [.11, .19] | .12 [.08, .16] |
| Non-Hispanic Black Female | .09 [.05, .13] | .11 [.06, .17] | .11 [.07, .16] | .09 [.06, .12] | .10 [.06, .15] |

*Note*. ST: standard hours; VH: variable hours; NW: not working. Numbers represented predicted probabilities of reporting depressive symptoms based on regression results reported in Table 2-2 with 95% confidence intervals shown in brackets.
